# SARS-CoV-2 NSP8-Derived Peptide Effectively Suppresses the Activity of Helicase NSP13

**DOI:** 10.34133/csbj.0174

**Published:** 2026-08-03

**Authors:** Shina Pashova, Peicho Petkov, Rositsa Hristova, Elena Krachmarova, Genoveva Nacheva, Anastas Gospodinov, Elena Lilkova, Nevena Ilieva, Miroslav Rangelov, Nadezhda Todorova, Anastas Pashov, Leandar Litov

**Affiliations:** ^1^ Institute of Biology and Immunology of Reproduction “Acad. K. Bratanov”, Bulgarian Academy of Sciences, Sofia 1113, Bulgaria.; ^2^ Sofia University “St. Kl. Ohridsky”, Physics Faculty, Sofia 1164, Bulgaria.; ^3^ Institute of Molecular Biology “Acad. Roumen Tsanev”, Bulgarian Academy of Sciences, Sofia 1113, Bulgaria.; ^4^ Institute of Information and Communication Technologies, Bulgarian Academy of Sciences, Sofia 1113, Bulgaria.; ^5^ Centre of Excellence in Informatics and Information and Communication Technologies, Sofia 1113, Bulgaria.; ^6^ Institute of Organic Chemistry with Centre of Phytochemistry, Bulgarian Academy of Sciences, Sofia 1113, Bulgaria.; ^7^ Institute of Biodiversity and Ecosystem Research, Bulgarian Academy of Sciences, Sofia 1113, Bulgaria.; ^8^ “Stephan Angeloff” Institute of Microbiology, Bulgarian Academy of Sciences, Sofia 1113, Bulgaria.

## Abstract

Severe acute respiratory syndrome coronavirus 2 (SARS-CoV-2) nonstructural protein 13 (NSP13) is a multifunctional helicase that plays a central role in viral RNA replication and suppression of host innate immunity. Beyond its essential function within the replication–transcription complex (RTC), NSP13 antagonizes type I interferon (IFN-I) signaling through interaction with the host kinase TBK1, making its protein–protein interactions attractive antiviral targets. In this study, we investigated whether fragments derived from the viral cofactor NSP8 can competitively interfere with NSP13 interactions. Using molecular dynamics simulations, interaction mapping, and neural network-based binding free energy prediction, we characterized NSP13 interfaces with (a) full-length NSP8 in 2 RTC binding modes, (b) N- and C-terminal NSP8 fragments, and (c) TBK1. Structural analyses revealed that the N-terminal fragment of NSP8 (NSP8-N, residues 1 to 87) binds NSP13 with high affinity and occupies residues critical for both TBK1 association and canonical NSP8–NSP13 interactions, including Met^68^, Tyr^93^, Phe^90^, Gly^67^, Ser^350^, and Thr^351^. Predicted binding free energies indicate that NSP8(N) forms a more stable complex with NSP13 than either the native RTC configuration or the NSP13–TBK1 complex, supporting a competitive binding mechanism. Functionally, coexpression experiments in poly(I:C)-stimulated A549 cells showed that, while NSP13 suppresses IFN-β transcription, coexpression of NSP8 or its N-terminal domain restores IFN-β levels to those of control cells. These findings support a dual inhibitory model in which NSP8-derived peptides sequester NSP13, preventing immune suppression and potentially impairing its recruitment to the RTC, highlighting the NSP8 N-terminal α-helical region as a promising scaffold for antiviral development.

## Highlights

• NSP8-N binds NSP13, occupying residues required for TBK1 and NSP8-NSP13 association.
• NSP8-N blocks NSP13-TBK1, occupies RTC, and inhibits immune evasion and replication.
• NSP8-N restores the IFN-β expression in cells coexpressing NSP13.

## Graphical Abstract

**Figure:**
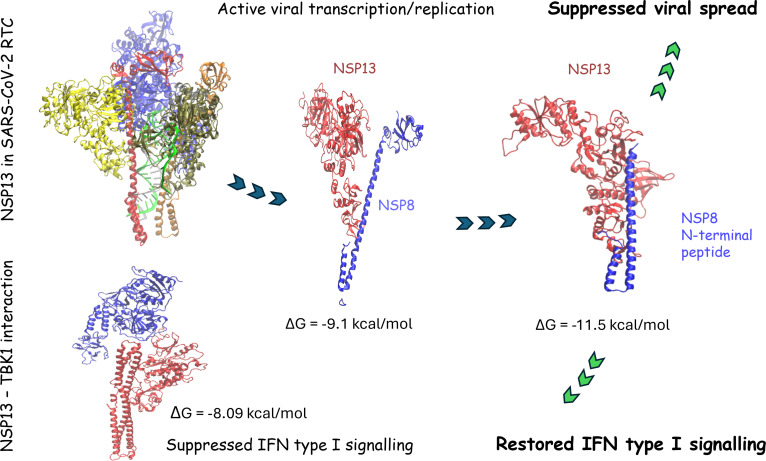

## Introduction

The global pandemic caused by the novel coronavirus severe acute respiratory syndrome coronavirus 2 (SARS-CoV-2) has spurred an unprecedented scientific response, leading to the rapid development of vaccines, diagnostics, and therapeutics. These advances have substantially mitigated the global burden of COVID-19. However, despite these efforts, the continued evolution of viral variants—particularly those belonging to the Omicron lineage—has led to reduced vaccine efficacy, primarily due to the high density of mutations localized in the spike (S) glycoprotein, the principal target of neutralizing antibodies [1–5]. The rapid antigenic drift of the spike protein underscores the need for identifying more genetically stable viral targets for long-term therapeutic intervention.

In contrast to the spike protein, many of SARS-CoV-2’s nonstructural proteins (NSPs), which are responsible for viral genome replication, transcription, and immune evasion, exhibit significantly lower mutation rates [1,6–10]. These NSPs are translated from the ORF1a/ORF1ab polyproteins encoded in the 5′ region of the 30-kb positive-sense viral RNA genome. The polyprotein is co- and post-translationally cleaved by viral proteases into 16 mature NSPs, which play distinct roles in cell invasion with several assembling into the replication–transcription complex (RTC) [11,12]. This complex localizes to double-membrane vesicles (DMVs) derived from the endoplasmic reticulum and mediates the synthesis of full-length and subgenomic viral RNAs.

Among the RTC components, nonstructural protein 13 (NSP13) stands out due to its conserved structure and multifunctionality. It is an SF1B-type helicase that exhibits 5′ to 3′ RNA unwinding activity in an adenosine triphosphate (ATP)-dependent manner, essential for viral RNA replication and transcription [9,13,14]. NSP13 also possesses RNA 5′-triphosphatase activity, which is involved in RNA capping, a critical step for the translation and stability of viral mRNAs [13]. Structurally, NSP13 consists of 5 domains: an N-terminal zinc-binding domain (ZBD), a stalk domain, a 1B domain, and 2 RecA-like domains (1A and 2A) that house the helicase active site.

In addition to its canonical role in RNA metabolism, NSP13 has been implicated as a potent antagonist of the type I interferon (IFN-I) response, a central axis of innate antiviral immunity [15–18]. One of the key pathways targeted by NSP13 is the TBK1–IRF3 axis. Normally, upon detection of viral RNA by cytosolic sensors such as RIG-I and MDA5, a signaling cascade that activates TANK-binding kinase 1 (TBK1) is triggered. Activated TBK1 phosphorylates interferon regulatory factor 3 (IRF3), which then dimerizes and translocates to the nucleus to induce IFN-β gene expression. However, NSP13 can directly interact with TBK1, thereby blocking IRF3 phosphorylation, preventing its nuclear translocation, and ultimately suppressing IFN-β transcription [18–20]. This inhibition allows the virus to evade early immune detection and establish a more permissive environment for replication. Moreover, a recent study revealed that the SARS-CoV-2 NSP13 modulates host immune responses by influencing miR-146a signaling pathways. Specifically, NSP13 up-regulates miR-146a, which in turn suppresses key components of the nuclear factor κB (NF-κB) pathway, potentially dampening inflammatory responses and aiding viral replication [21].

Although NSP13 is considered one of the most conserved proteins among coronaviruses [9], computational studies have revealed rare mutations in this protein that may enhance its ability to antagonize the immune response [20]. These findings underscore the adaptive potential of SARS-CoV-2 even within structurally constrained regions of its proteome and highlight the need for targeted strategies that can disrupt its function without relying solely on mutational stability.

**Fig. 1.**
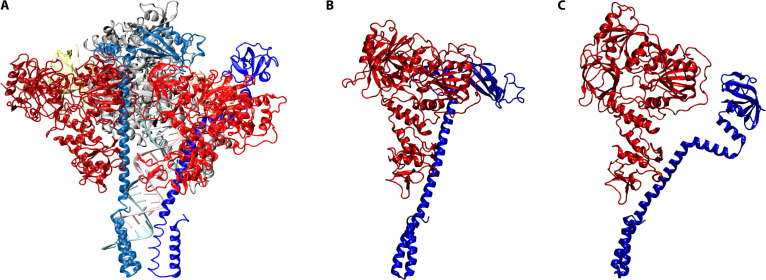
(A) SARS-CoV-2 RTC. The 2 NSP13 molecules and the 2 NSP8 molecules are shown in shades of red and blue, respectively; the dsRNA fragments are colored light pink and cyan, respectively. The RNA-dependent RNA polymerase (RdRp) is shown in gray, while the cofactors NSP7 and NSP9 are shown in orange and yellow, respectively. (B) NSP8 (blue)–NSP13 (red) binding mode I in the RTC (chains B and E). (C) NSP8 (blue)–NSP13 (red) binding mode II in the RTC (chains D and F). Complexes (B) and (C) are aligned by NSP13.

Understanding the interactions between NSP13 and its viral cofactors is crucial for identifying novel therapeutic strategies that disrupt its dual role in viral RNA replication and immune evasion. Within the RTC (Fig. 1A), NSP13 does not act in isolation. Instead, it operates in concert with NSP12 (RNA-dependent RNA polymerase) and cofactors such as NSP7 and NSP8, forming a dynamic multi-protein machinery responsible for genome duplication and subgenomic RNA synthesis [11,12]. Among these, NSP8 plays a critical role as a structural adaptor, facilitating both enzymatic activity and recruitment of additional NSPs to the RTC.

NSP8 is characterized by a long N-terminal α-helix, which has been shown through cryo-electron microscopy (EM) and x-ray crystallography to directly interact with the ZBD of NSP13 [22,23]. This interaction forms a stable interface that not only positions NSP13 correctly within the RTC but also modulates its helicase function [12,23]. Notably, 2 NSP13 molecules can associate simultaneously with the NSP12–NSP8–NSP7 complex, forming an asymmetric architecture (Fig. 1B and C) in which each NSP13 is functionally integrated at distinct sites of the RTC [23]. Disrupting this interface could effectively displace NSP13 from its active role in replication or suppress its helicase activity.

Targeting this protein–protein interaction (PPI) offers a strategically valuable therapeutic window, distinct from conventional enzymatic inhibitors. Unlike catalytic domains that tend to be flexible and difficult to drug selectively, protein interaction surfaces are often more specific and accessible, particularly when involving structural adaptors like NSP8. Furthermore, because the NSP13–NSP8 interface is essential for RTC stability and is highly conserved among β-coronaviruses, it represents a robust antiviral target unlikely to succumb to rapid mutational escape.

Recent studies [9,24] have categorized potential helicase inhibitors based on their binding sites, including compounds targeting the ZBD, the nucleic acid-binding site, the nucleotide-binding site, and other allosteric or uncharacterized pockets. Molecules such as ebselen [25], flavonoids [26], bananins [27], and clofazimine [28] have shown varying degrees of inhibition on helicase functions like RNA unwinding and adenosine triphosphatase (ATPase) activity. These findings underscore the potential of targeting NSP13 beyond its catalytic core, highlighting the importance of structural dynamics and allosteric regulation in future drug design.

We aimed to identify potential NSP13-binding sites that could be exploited to disrupt the NSP13–NSP8 interaction. Using in silico docking and molecular dynamics (MD) simulations, we mapped the interface between these 2 proteins, confirming the N-terminal α-helix of NSP8 and the zinc finger–containing N-terminal domain of NSP13 as critical contact points [29]. These computational results aligned with existing structural data, validating our strategy of developing a peptidomimetic fragment derived from NSP8 capable of selectively binding NSP13.

We hypothesize that an exogenous NSP8-derived peptide, encompassing only the key interface-forming residues, could act as a dominant-negative inhibitor. By competing with full-length NSP8 for binding to NSP13, this fragment would sequester NSP13 in a nonfunctional complex, preventing its incorporation into the RTC. Additionally, such binding might obstruct NSP13’s ability to interact with host proteins such as TBK1, thereby reducing its immunosuppressive effects. Importantly, because NSP13’s interaction with NSP8 is specific to the viral replication machinery, targeting this interface may offer greater selectivity than small-molecule ATPase inhibitors that target a conserved enzymatic site, although potential off-target interactions with host proteins cannot be excluded and would require thorough experimental evaluation.

To test this hypothesis, we performed MD simulations of the interactions between NSP13 and TBK1, as well as between NSP13 and NSP8, and investigated their potential binding sites. Additional simulations were carried out using fragments of NSP8 containing either the C-terminal or N-terminal regions, in complex with NSP13. The results of these simulations indicated that the N-terminal fragment of NSP8 binds to NSP13 in a manner that prevents its normal incorporation into the RTC and simultaneously blocks its interaction with TBK1. To validate the central hypothesis suggested by our in silico findings, we engineered an expression system in A549 human lung epithelial cells, wherein we assessed IFN-β promoter activation under conditions of NSP13 overexpression alone and in the presence of coexpressed NSP8 fragments. The experimental data support our hypothesis: NSP13 alone reduces IFN-β induction, consistent with its known inhibitory role, whereas coexpression with specific NSP8 domains partially restores IFN signaling, suggesting successful sequestration or functional inactivation of NSP13.

## Materials and Methods

### Molecular modeling

#### MD simulations

Initial atomic models of NSP8 and NSP13 and truncated versions of NSP8 were derived from the cryo-EM structure of the SARS-CoV-2 RTC, deposited under Protein Data Bank (PDB) ID 7CYQ [23]. This structure (shown in Fig. 1A) was selected for its complete representation of full-length NSPs in complex within the RTC. Chain B was used as the template for NSP8, while chain E was selected for NSP13. A structural comparisson of the available structures of NSP8 is shown in Fig. S1. Root mean square deviation (RMSD) of the backbone of amino acid residues 10 to 190 is ∼1.2 ± 0.5 Å.

Truncation of NSP8 was performed at the junction between residues Met^87^ and Gln^88^ to yield N- and C-terminal fragments. To assess the robustness of the predicted binding mode, additional simulations were performed using the experimentally resolved structure of the NSP8 N-terminal domain (PDB ID: 7YWR) [30] in complex with NSP13. For simulations involving TBK1, the initial atomic model was taken from the crystal structure deposited in the Protein Data Bank under accession code PDB ID 4IWO [31]. All protein models were pre-equilibrated via short MD simulations of 20 ns.

All simulations were performed using GROMACS (v2020.1 and newer) [32,33]. Protein topologies were generated using the CHARMM36m force field [34]. The structural Zn_2+_ center of the NSP13 ZBD was modeled using the CYM residue parameters from the March 2019 CHARMM36 force field release. The topology was modified by incorporating the Zn_2+_ ion into the CYM residue definition and introducing explicit Zn–S covalent bonds to the coordinating cysteine SG atoms, thereby preserving the experimentally observed tetrahedral coordination throughout the simulations.

For the protein–protein binding simulations, the binding partners were initially placed approximately 2 nm apart and allowed to bind spontaneously during the MD simulations. Each protein or complex was solvated in a cubic simulation box filled with explicit modified TIP3P water and a minimum solute–wall distance of 2.0 nm. Sodium and chloride counterions were added to neutralize the system and to reproduce a physiological ionic strength of 0.15 M. Energy minimization was performed using steepest-descent algorithm until the maximum force was reduced below 100 kJ. mol^−1^. nm^−1^. This was followed by a 2-stage equilibration protocol: 1 ns of constant-volume (NVT) simulation using a velocity-rescale thermostat at 310 K [35], and 10 ns of isothermal-isobaric (NPT) equilibration using the Parrinello–Rahman barostat at 1-bar pressure [36].

Production trajectories were propagated using the leap-frog integrator with a 2-fs time step, and constraints on all hydrogen-involving bonds were applied using the PLINCS algorithm [37]. Temperature was maintained at 310 K using the v-rescale thermostat (coupling constant 0.1 ps) and pressure at 1 atm using the Parrinello–Rahman barostat (coupling constant 2.0 ps). Electrostatics were calculated via the smooth particle-mesh Ewald method (PME) [38] with a real-space cutoff of 1.2 nm. Van der Waals interactions were truncated at 1.2 nm with a smooth switching function starting at 1.0 nm. MD simulations were run for 350 to 550 ns, depending on the system, with coordinates recorded every 100 ps.

The MD trajectories were post-processed and analyzed using the standard GROMACS analysis tools to calculate the RMSD, root mean square fluctuation (RMSF), and structural clusters. Prior to all analyses, each trajectory was least-squares fitted to the initial conformation of NSP13 to remove global translational and rotational motions and align the complexes with respect to the protein of interest. Structural clustering was performed using the GROMOS algorithm [39]. All structural figures were generated using the molecular visualization and analysis package VMD [40].

#### Protein–protein interaction analysis

Protein–protein contact maps were generated using LIGPLOT+ [41,42], which automatically identifies hydrogen bonds and hydrophobic contacts from structural coordinates and produces 2-dimensional (2D) schematic representations of intermolecular interactions. Dimpyplot_chordplot was used to batch-process the largest cluster conformations of the complexes with LIGPLOT+.

#### Estimation of the binding free energy

A 3D convolutional neural network (CNN) was constructed to estimate the binding free energy of protein–peptide complexes. The model takes as input a 3D grid of size 80 × 80 × 80, in which each voxel corresponds to a discrete spatial location. The center of each amino acid residue is encoded using a categorical label representing one of 20 receptor residue types or one of 20 ligand residue types, allowing the network to distinguish residues originating from the 2 interacting molecules.

The proposed architecture comprises 2 parallel convolutional branches that independently learn hierarchical spatial representations. Each branch starts with a 3D convolutional layer containing 20 filters and a kernel size of (2,2,2), followed by a pooling operation that reduces the dimensionality of the feature maps while preserving their most informative characteristics. One branch applies max pooling, whereas the other uses average pooling, enabling the extraction of complementary spatial features. Subsequently, each branch includes 5 additional 3D convolutional layers with 10 filters each, followed by a second pooling layer that further compresses and refines the learned representations.

The feature maps produced by the 2 branches are flattened and merged into a single feature vector, which is then processed by a sequence of fully connected layers containing 8, 4, and 2 neurons, respectively. This gradual reduction in dimensionality promotes generalization and helps limit overfitting. A final output neuron predicts the binding free energy as a single continuous value. Model optimization was performed with the Adam algorithm using the mean squared error (MSE) as the loss function, while the mean absolute error (MAE) served as an additional measure of predictive performance.

By combining max pooling and average pooling within parallel branches, the network captures complementary aspects of the spatial organization of protein–peptide interfaces, thereby improving the prediction of binding free energies. The model was trained and evaluated on 183 protein–peptide complexes obtained from the PROXiMATE database [43], with 150 complexes used for training and the remaining 33 reserved for validation. The resulting model achieved a validation MSE of 7.09 (kcal/mol) and an MAE of 1.93 kcal/mol. This CNN has previously been applied successfully to predict the effects of point mutations in the receptor-binding motif of the SARS-CoV-2 spike protein on its interaction with the human ACE2 receptor [44].

In addition, the conformations in the largest clusters for the respective complex were used to estimate the binding free energies using 4 different external methods. We performed molecular mechanics generalized Born surface area (MM/PBSA) calculations using gmx_MMPBSA [45,46]. The PRODIGY (PROtein binDIng enerGY prediction) model [47–49] uses interfacial contacts, non-interacting surface, and machine learning (ML) and empirical models to predict the binding affinity of biomolecular complexes directly from their 3D structures. PBEE (protein binding energy estimator) [50] is an ML ensemble model that uses Rosetta-based quantities to predict binding free energies of protein–protein complexes from their PDB coordinates. PPB-Affinity (protein–protein binding affinity) [51] is a comprehensive dataset and a benchmark algorithm based on geometric deep learning method that uses the invariant point attention method to extract features from the crystal structure of protein–protein complexes to predict the affinity magnitude.

### Experimental measurements

#### Cell culture

A549 cell line was a kind gift from the Laboratory “Experimental Immunotherapy”, Department of Immunology at Institute of Microbiology, BAS, Sofia, Bulgaria. Cells were grown in Dulbecco’s modified Eagle’s medium (DMEM), high glucose (Gibco, ref: 11965-092) supplemented with 10% fetal bovine serum (FBS; Gibco, ref: 10437-028) and 1% penicillin/streptomycin (Gibco, ref: 15140-122). Stimulation of the endogenous IFN-β production was achieved by using 2 μg/ml synthetic double-stranded RNA (dsRNA) polyinosinic:polycytidylic acid [poly(I:C); InvivoGen, #tlrl-picw]. The cells were maintained in humidified incubator at 37 °C with 5% CO_2_.

#### Plasmids

The plasmids encoding NSP8 (#157694), NSP13 (#157714), and the empty vector (EV; #19446) were obtained from Addgene. The original expression vector for NSP8 was re-engineered (Eurofins) into 2 distinct plasmids: one encoding the N-terminal domain (NSP8-N; residues 1 to 87) and the other encoding the C-terminal domain (NSP8-C; residues 88 to 198). Plasmids were transformed and propagated into *Escherichia coli* TOP10 strain. Plasmids were isolated using a megaprep kit (Qiagen, #12181) and verified by restriction analysis.

#### Transfection

The day before transfection, cells were seeded at 4 × 10^5^ cells per well in a 6-well plate in supplemented DMEM and incubated overnight. The next day, the medium was changed to Opti-MEM (Gibco, #31985070) and cells were transfected with poly(I:C) (2 μg/ml) and plasmids (3.2 μg/well, equalized to this amount with EV where needed) using Lipofectamine 3000 (Invitrogen, #L3000-008) according to the manufacturer’s protocol. The transfection mix was removed after 20 h, and cells were collected for further analysis.

#### RNA isolation and RT-qPCR

Total RNA was extracted from A549 cells treated with poly(I:C) and transfected with EV, full-length or domain-specific NSP8 constructs, and NSP13, using the RNeasy Plus Kit (Qiagen, #74134), following the manufacturer’s protocol. The concentration and purity of extracted RNA were determined by NanoDrop-1000 (Thermo Fisher, Waltham, MA, USA). RNA integrity and quality were assessed using 1% agarose gel electrophoresis. Total RNA (1 μg) from each sample was reverse transcribed by RevertAid H Minus First Strand cDNA Synthesis Kit (Thermo Scientific, Waltham, MA, USA, catalog number K1632). The relative expression levels of the target hIFN-β gene were assessed by a real-time quantitative polymerase chain reaction (RT-qPCR) analysis using the Maxima SYBR Green qPCR Master Mix (Thermo Scientific). The housekeeping glyceraldehyde-3-phosphate dehydrogenase (GAPDH) gene was used as an endogenous control to normalize gene expression. The target and housekeeping genes were amplified with the primers shown in Table 1.

**Table 1.** Primer sequences used for RT-qPCR analysis of IFN-β expression

Gene	Sequence
IFN-β	Forward 5′-CTTGGATTCCTACAAAGAAGCAGC
	Reverse 5′-TCCTCCTTCTGGAACTGCTGCA
GAPDH	Forward 5′-GTCTCCTCTGACTTCAACAGCG
	Reverse 5′-ACCACCCTGTTGCTGTAGCCAA

The analysis was performed on a Rotor-Gene 6000 thermal cycler (Corbett, QIAGEN, Hilden, Germany). Gene expression data were analysed using Rotor-Gene 6000 Software (QIAGEN), and the relative expression levels of the hIFN-β gene were normalized to the endogenous control for each sample. Each RT-qPCR was performed in at least 3 replicates in different PCR runs. Statistical differences between the different transfection conditions were evaluated using analysis of variance (ANOVA), and *P* values <0.05 were considered statistically significant.

## Results

### In silico investigations

**Fig. 2.**
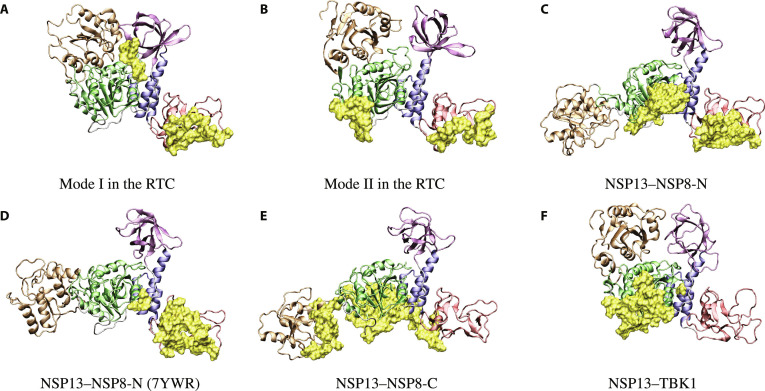
Binding interfaces on the conformation of the centroid of the helicase in the different binding MD simulations. NSP13 binding to (A) NSP8 in the RTC, mode I; (B) mode II; (C) N-terminal fragment of NSP8; (D) NSP8-N starting from the 7YWR conformation; (E) C-terminal fragment of NSP8; and (F) TBK1. The NSP13 protein domains are colored as follows: ZBD (amino acids 1 to 99) in red; Stalk (amino acids 102 to 150) in blue; 1B (amino acids 151 to 229) in purple; Rec1A (amino acids 260 to 442) in green; Rec2A (amino acids 443 to 596) in orange; and loops in white. The binding interface, identified based on the LIGPLOT contacts maps, is shown in yellow.

All interaction analyses presented in this study are based on representative conformations extracted from extensive MD simulations through clustering of the trajectories, as described in the MD simulations and Estimation of the binding free energy sections. Results from the clustering procedure are presented in Fig. S2. Each NSP13–protein complex was docked and refined using unbiased MD simulations for spontaneous binding. The resulting stabilized structures were used to generate LIGPLOT+ interaction maps, ensuring that only biologically relevant, energetically favorable contacts were considered. The obtained contact maps are shown in Figs. S3 to S8. Based on them, the binding interfaces on the surface of the helicase were constructed (Fig. 2). Structural metrics for all systems, such as RMSF and RMSD, are provided in Figs. S9 and S10.

#### Structural insights into the binding of the NSP8 N-terminal peptide to NSP13

This investigation aimed to determine the precise structural interface, assess domain-specific engagement, and evaluate whether such binding could interfere with the normal incorporation of NSP13 into the SARS-CoV-2 RTC or its interactions with host immune regulators.

**Fig. 3.**
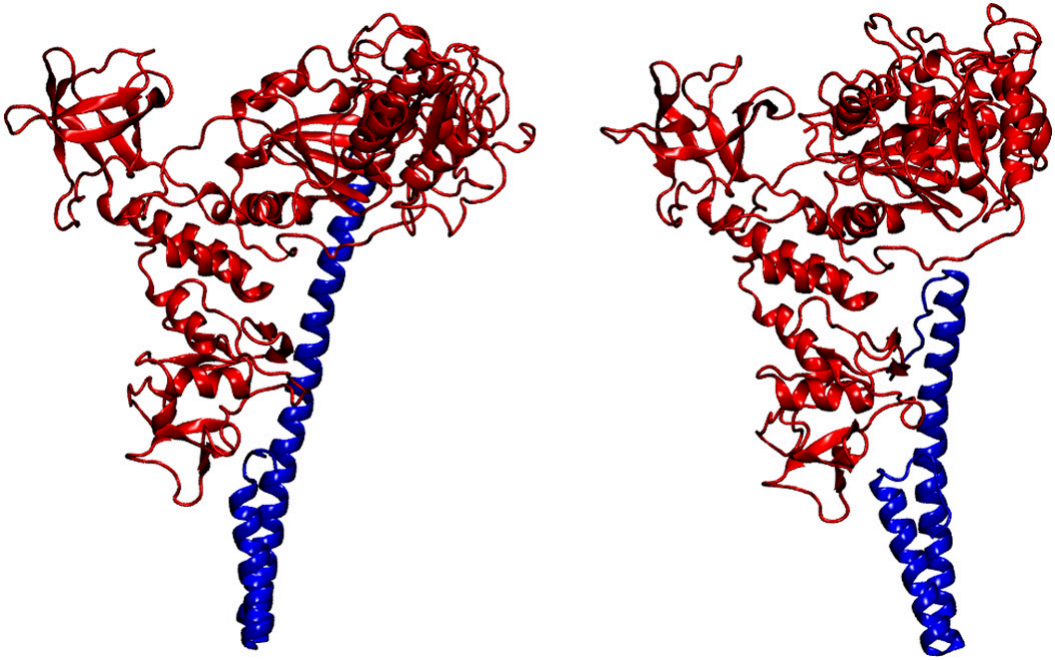
Representative conformations of the NSP8-N (blue)–NSP13 (red) complex.

The contact maps from the LIGPLOT analysis (Fig. S5) reveals that a peptide fragment from NSP8 (residues 1 to 87) engages in a network of specific hydrogen bonds and hydrophobic interactions with residues of NSP13. The centroid of the largest cluster in this trajectory is presented in Fig. 3. Key hydrogen bonds were identified between Asp^78^ and Arg^392^, Met^87^ and Ser^350^, and Ser^85^ multiple residues—Thr^351^, Thr^367^, and Arg^303^. These bonds likely play a critical role in stabilizing the interface. A salt bridge was observed between Arg^80^ (NSP8-N) and Glu^365^ (NSP13), providing strong electrostatic anchoring.

Our simulations reveal that the NSP8-N fragment binds primarily to the ZBD of NSP13, which comprises the first 60 residues of the helicase. Key residues from ZBD, such as Val^2^, Gly^67^, Met^68^, Ile^79^, Phe^81^, Tyr^93^, and Lys^S94^, form a tight hydrophobic and polar interaction network with residues from NSP8 including Leu^9^, Pro^10^, Ser^11^, Asp^52^, Met^55^, Arg^57^, Ala^63^, Ala^66^, Met^67^, Glu^77^, Ala^81^, Tyr^84^, Ser^85^, and Met^87^. The resulting interface is stabilized by aliphatic stacking, aromatic–polar contacts, and hydrogen bonds, suggesting a high degree of specificity and stability in the binding.

Importantly, in addition to this ZBD-centered core, the NSP8-N fragment also engages more distal sites on NSP13, particularly through its C-terminal region near Met^87^. This residue forms stabilizing interactions with residues Lys^345^, Phe^343^, Asp^344^, Thr^351^, and Ser^350^, which are located in the RecA2 helicase domain of NSP13. These interactions expand the contact surface and may interfere with functionally relevant surfaces of NSP13, such as those required for RNA unwinding or host factor binding (Fig. 2C).

A simulation of the binding of experimentally resolved structure of the C-terminal domain of NSP8 with PDB ID 7YWR (Fig. 3) (amino acids 1 to 84) displays similar binding mode, although somewhat different residues are engaged in the complex. The contact map of this system is shown in Fig. S6. Overall, the Nsp13–Nsp8-N interaction interface is largely conserved (Fig. 2C and D), dominated by hydrophobic interactions in several well-defined clusters.

#### Interaction analysis of the C-terminal fragment of NSP8 with NSP13

Detailed analysis of the residue-level contacts (see Fig. S7) reveals that the interaction between the C-terminal region of NSP8 and NSP13 is organized into 3 spatially distinct interaction clusters, each involving a combination of hydrophobic and polar contacts. Together, these clusters form a stable and extended binding interface, indicating strong affinity of the NSP8 C-terminal fragment for NSP13 while remaining topologically distinct from the canonical NSP8–NSP13 binding surface observed in the RTC (Fig. 2A, B, and E).

**Fig. 4.**
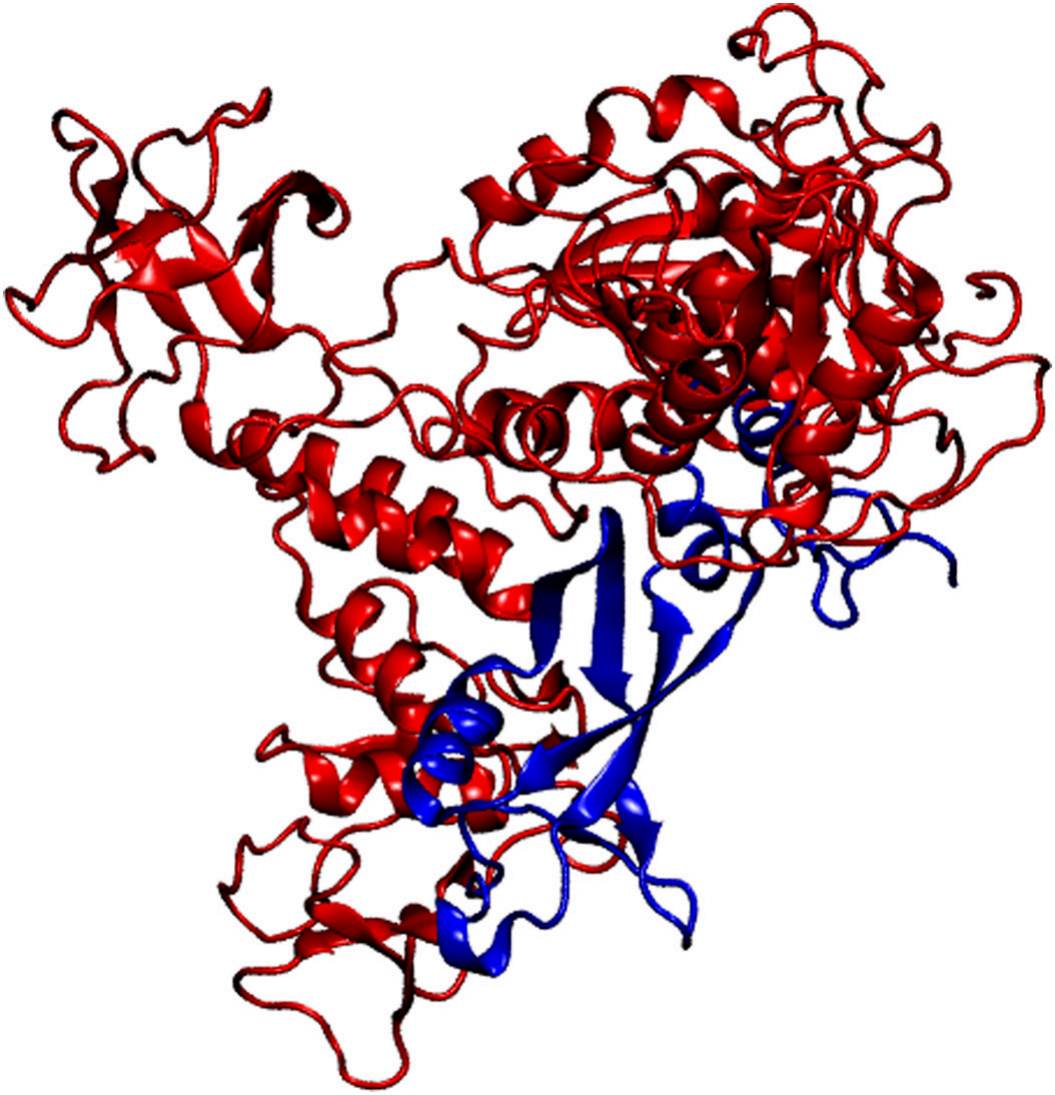
Representative conformation of the NSP8-C (blue)–NSP13 (red) complex.

The interaction pattern shown reflects a representative conformation from the MD trajectory (Fig. 4), supporting the hypothesis that the C-terminal domain of NSP8 engages a distinct and functionally relevant surface of NSP13, complementary to the N-terminal interface.

The first interaction cluster involves residues Leu^90^ to Leu^95^ of NSP8-C, which predominantly engage residues Tyr^457^ to Leu^461^, Leu^581^, and Lys^584^ of NSP13. This region is characterized mainly by hydrophobic and aromatic interactions, including 𝜋–𝜋 stacking between aromatic side chains and van der Waals contacts between aliphatic residues. The close packing of Phe^92^, Met^94^, and Leu^95^ against Tyr^457^ and Asp^458^ suggests the formation of a compact hydrophobic patch that contributes significantly to the stability of the complex.

A second interaction cluster spans residues Ala^102^ to Ile^120^ of NSP8-C, engaging a broader surface region of NSP13 encompassing residues Gln^243^ to Gln^275^ and Ile^432^ to Lys^460^. This interface combines polar interactions, hydrogen bonding, and electrostatic contacts, particularly involving charged residues such as Asp^112^ and Arg^111^ from NSP8-C and Lys^460^ from NSP13. The mixed polar–hydrophobic nature of this region suggests a flexible but stabilizing interface, likely allowing conformational adaptability while maintaining strong binding.

The third interaction cluster involves residues Cys^142^ to Glu^171^ of NSP8-C, which interact with residues Lys^28^ to Asp^105^ of NSP13. This region is dominated by polar interactions and hydrogen bonds, with contributions from backbone and side-chain contacts. The presence of cysteine and acidic residues in this cluster supports a role for directional hydrogen bonding and dipole–dipole interactions rather than purely hydrophobic packing.

Importantly, although the C-terminal fragment of NSP8 binds strongly to NSP13 through these 3 interaction clusters (see Fig. 2E), the binding surface is distinct from the region utilized by the N-terminal domain of NSP8 for NSP13 engagement within the RTC (Fig. 2A and B). Consequently, this interaction is not expected to sterically or competitively block the canonical NSP8–NSP13 association. Instead, the C-terminal NSP8–NSP13 interface appears to represent an auxiliary or stabilizing contact surface, potentially contributing to the structural organization or regulation of NSP13 within the multi-protein replication machinery.

#### Interaction of NSP13 with TBK1: Structural characterization of the binding Interface

**Fig. 5.**
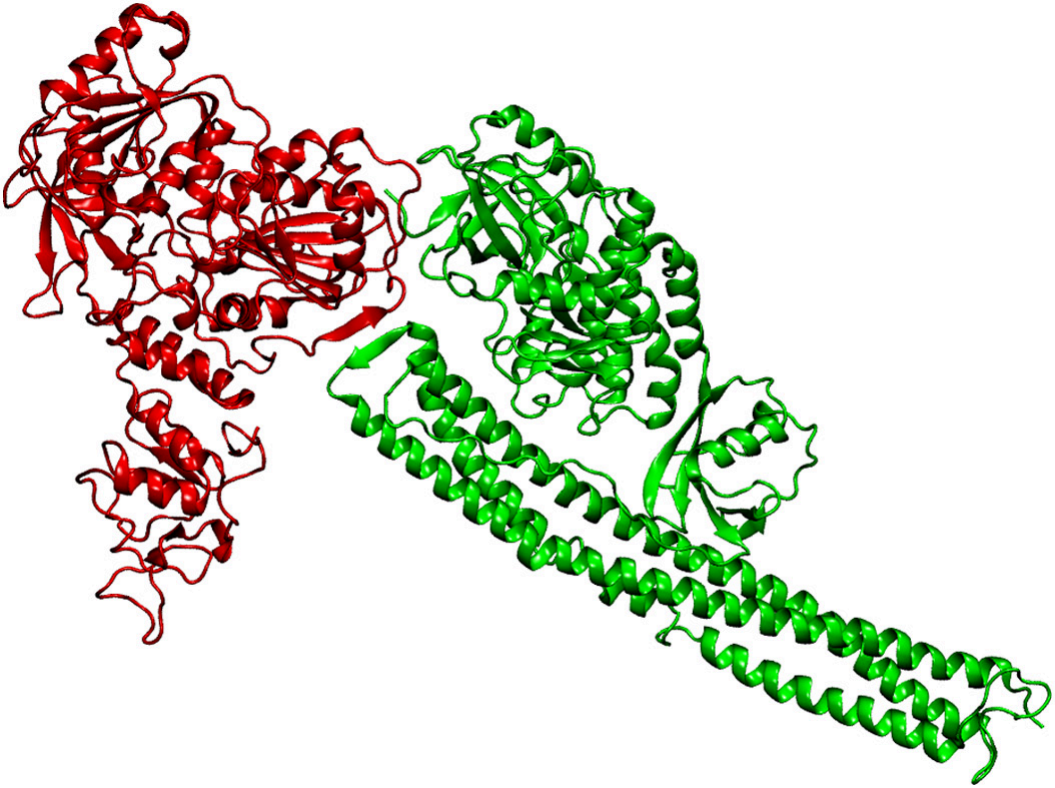
Representative conformation of the NSP13 (red)–TBK1 (green) complex.

Analysis of the NSP13–TBK1 complex (Figs. 2F and 5) reveals a well-defined protein–protein interaction interface stabilized by a combination of hydrogen bonds, electrostatic interactions, polar contacts, and hydrophobic packing. The full contact map is presented in Fig. S8. The interaction surface is predominantly formed by residues from the N-terminal and central regions of NSP13 engaging a continuous surface on TBK1, suggesting a stable and functionally relevant association.

Several strong hydrogen bonds anchor NSP13 to TBK1. Notably, Glu^244^, His^245^, Val^247^, Ile^249^, and Thr^250^ (NSP13) form hydrogen bond with Ala^574^–Leu^578^ (TBK1), which are further reinforced by multiple short-range nonbonded contacts between the same residue pair, effectively constituting a persistent salt bridge-like interaction. Additional hydrogen bonds involve Arg^577^ (TBK1) interacting with Glu^244^ (NSP13), and Tyr^580^ (TBK1) forming contacts with His^245^ (NSP13). These interactions indicate that charged and polar residues play a dominant role in defining the specificity and strength of the NSP13–TBK1 interface.

The repeated involvement of Arg^576^ and Arg^577^ (TBK1) in both hydrogen bonding and electrostatic proximity to acidic residues on NSP13 highlights a locally charged interaction hotspot. Such clusters are characteristic of regulatory protein–protein interfaces and are consistent with a role in signaling interference rather than transient, nonspecific binding.

Beyond polar interactions, the interface is further stabilized by extensive hydrophobic contacts, particularly involving Gly^1^–Gln^5^ and Leu^13^–Ile^17^ of TBK1, which pack against hydrophobic and aromatic residues of NSP13 such as Tyr^253^–Leu^256^, Ser^301^, and Val^348^–Gln^354^. These interactions form a hydrophobic patch that likely contributes to the longevity of the complex in solution. Aromatic residues also participate in 𝜋-type interactions, exemplified by contacts between Tyr^580^ (TBK1) and the imidazole ring of His^245^ (NSP13). Such interactions are known to enhance binding specificity and orient protein domains relative to each other.

Importantly, the interaction surface on NSP13 involved in TBK1 binding overlaps with regions implicated in protein–protein interactions rather than with the catalytic helicase core. This spatial separation suggests that NSP13 binding to TBK1 does not directly inhibit helicase activity but instead exerts its effect through sequestration or steric interference with TBK1 function, consistent with the observed suppression of IRF3 phosphorylation reported in functional studies [19].

The NSP13–TBK1 interaction is characterized by a dense polar–electrostatic core flanked by hydrophobic stabilization regions, forming a robust interface well suited for sustained inhibition of host innate immune signaling (Figs. 2F and 5). The location and composition of this interface provide a structural basis for NSP13-mediated antagonism of the IFN-I pathway and establish a clear contrast with NSP13 interactions mediated by viral cofactors such as NSP8.

#### Comparative mapping of NSP8-N–NSP13 and NSP13–TBK1 interfaces

To evaluate whether the NSP8 N-terminal fragment engages NSP13 at a surface relevant for TBK1 recognition, we performed a direct structural comparison of the MD-derived NSP8-N–NSP13 and NSP13–TBK1 (Fig. 2C and F) complexes. Structural superposition revealed a clear spatial overlap on the NSP13 surface. Several residues central to TBK1 binding, including Glu^244^, His^245^, Tyr^246^, Val^247^, Ser^350^, and Glu^353^, were also found to participate in stable interactions with the NSP8 N-terminal fragment. In particular, the NSP8 peptide spans the ZBD-proximal corridor and extends toward the Ser^350^–Thr^351^–Glu^353^ region, which forms part of the polar–electrostatic core of the TBK1 interface. Residues such as Ser^350^ and Glu^353^, which contribute to hydrogen bonding and electrostatic stabilization in the NSP13–TBK1 complex, are likewise engaged by NSP8-N. Additionally, contacts involving Phe^343^, Asp^344^, Lys^345^, and Thr^351^ further consolidate occupation of this surface. The resulting binding footprint partially coincides with the charged hotspot defined by the TBK1 Arg-rich region.

In contrast, the C-terminal region of NSP8 binds NSP13 at a distinct helicase-associated surface around Tyr^253^–Pro^254^–Leu^256^, without overlap with the TBK1-binding interface. This structural segregation distinguishes the 2 NSP8-derived binding modes.

#### Distinct binding modes of NSP8 to NSP13 within the RTC complex

SARS-CoV-2 NSP13 is recruited to the viral RTC via dual interaction sites with NSP8. Cryo-EM data reveal that 2 molecules of NSP13 associate independently with NSP8–NSP12–NSP7 complex, forming asymmetric yet functionally significant interfaces [23]. To explore these interfaces at atomic resolution, we performed MD simulations followed by LIGPLOT+ interaction mapping.

##### Primary interface—N-terminal NSP8 helix anchoring

This interaction (Fig. 1B) involves NSP8 (chain B) engaging the N-terminal region of NSP13 (chain E), including residues from the ZBD and its immediate vicinity. Several transient hydrogen bonds contribute to directional stabilization of the interface and promote proper orientation (Fig. S3).

The dominant contribution to interface (Fig. 2A) stability arises from a dense cluster of hydrophobic and van der Waals interactions, indicating that binding mode I is primarily driven by nonpolar packing rather than extensive polar complementarity. A prominent hydrophobic patch is formed by Leu^92^, Tyr^93^, Phe^90^, and Val^45^ of NSP13, which pack against Tyr^71^, Met^70^, Gln^73^, and Glu^77^ of NSP8. In particular, Leu^92^ (NSP13) establishes multiple contacts with Tyr^71^ and Met^70^ (NSP8), suggesting tight aliphatic–aromatic packing. Similarly, Val^45^ (NSP13) interacts with Gln^73^ (NSP8) through its aliphatic side chain, reinforcing the hydrophobic core of the interface. Additional stabilization is provided by interactions between Met^68^ (NSP13) and Gln^69^ (NSP8), and between Phe^90^ (NSP13) and Met^70^ (NSP8), further extending the hydrophobic contact surface. The aromatic residues Phe^81^ and Tyr^48^ of NSP13 also participate in nonbonded contacts with Ala^63^ and Met^70^ of NSP8, contributing to 𝜋–alkyl and aromatic–aliphatic interactions. At the periphery of the interface, Ser^80^ and Ile^79^ of NSP13 engage Leu^59^ of NSP8 through backbone and side-chain contacts. Although these interactions are weaker individually, together they increase the contact area and reduce solvent exposure of the interface.

##### Secondary interface—Hydrophobic NSP8–NSP13 contacts

The second binding mode of NSP8 to NSP13 within the RTC (Fig. 1C) is characterized by a nonbonded interaction pattern, dominated by hydrophobic and aromatic contacts and a few direct hydrogen bonds (Fig. S4). This interface involves residues from the central helicase region of NSP13 (chain F) interacting with the C-terminal region of NSP8 (chain D), indicating a binding geometry distinct from the ZBD-associated binding mode I (Fig. 2B). A prominent feature of this interface is a strong aromatic–aliphatic interaction cluster centered on Tyr^253^ of NSP13, which establishes multiple close contacts with Pro^183^ of NSP8. All aromatic ring atoms of Tyr^253^ participate in van der Waals interactions with the proline side chain, forming a compact hydrophobic pocket. This extensive contact pattern suggests tight packing and a significant contribution to interface stability despite the absence of hydrogen bonding.

Additional stabilization is provided by interactions between Pro^254^ of NSP13 and Trp^182^ of NSP8, where backbone and side-chain atoms of Pro^254^ contact the indole ring of Trp^182^. These contacts represent aromatic–aliphatic and 𝜋–alkyl interactions that further reinforce the hydrophobic character of the interface. The involvement of consecutive residues Tyr^253^–Pro^254^ highlights a localized binding hotspot on NSP13. Peripheral hydrophobic contacts extend the interface toward adjacent regions. Leu^256^ of NSP13 interacts with Pro^178^ of NSP8, while Met^68^ and Gly^67^ of NSP13 contact Ala^63^ and Leu^59^ of NSP8, respectively. These interactions increase the buried surface area and reduce solvent exposure, collectively stabilizing the complex through distributed van der Waals forces.

#### Structural interference of NSP8-N with both NSP8–NSP13 RTC interfaces

To evaluate whether the NSP8 N-terminal fragment interferes with the native NSP8–NSP13 interactions within the RTC, we performed a residue-level comparative analysis across the corresponding complexes.

In the primary RTC interface (Fig. 2A), NSP13 residues Met^68^, Phe^81^, Phe^90^, and Tyr^93^ are central contributors to NSP8 binding. Structural comparison revealed that these same residues are engaged by NSP8-N in the NSP8-N–NSP13 complex. Tyr^93^ of NSP13 forms contacts with Ala^63^ and Asp^64^ of NSP8-N, Met^68^ interacts with Met^55^ and Pro^10^, and Phe^90^ engages Gln^56^ of NSP8-N. Since these residues are directly involved in ZBD-mediated anchoring of NSP13 to full-length NSP8 within the RTC, their occupation by NSP8-N indicates spatial overlap between the 2 binding modes.

A similar pattern is observed in the secondary RTC interface (binding mode II), which involves hydrophobic contacts mediated by Gly^67^ and Met^68^ of NSP13. In the NSP8-N–NSP13 complex, both Gly^67^ and Met^68^ establish stable interactions with Met^55^, Pro^10^, Asp^52^, and Ser^11^ of NSP8-N. Because Gly^67^ and Met^68^ contribute to stabilization of the second NSP8–NSP13 pair within the RTC, their engagement by NSP8-N indicates partial overlap with this interface as well.

These observations demonstrate that NSP8-N engages residues on NSP13 that are required for both canonical NSP8–NSP13 interaction modes within the RTC, revealing substantial structural overlap between the binding surfaces.

#### Estimation of binding free energy and affinity of competing NSP13

To quantitatively compare the stability and competitive potential of the investigated protein–protein complexes, we estimated the binding free energy (𝛥*G*) using the CNN described in Materials and Methods.

The predicted binding free energies (shown in Table 2) span a range of approximately 5 kcal/mol, indicating substantial differences in complex stability. Among the full-length NSP8–NSP13 interactions within the RTC, binding mode II (DF chains) exhibits a stronger interaction (𝛥*G* = −10.7 kcal/mol) compared to binding mode I (BE chains) (𝛥*G* = −9.1 kcal/mol), consistent with the more extensive hydrophobic packing observed in the second interface.

**Table 2.** Comparison of predicted binding energies of NSP13 complexes. Values are reported as mean ± standard deviation, with the rank shown in parentheses. CNN predictions are single-point estimates.

Complex	CNN	MM/PBSA	PRODIGY	PBEE	PPB
NSP8–NSP13 binding mode I	−9.1 ± 1.9 (5)	32.9 ± 2.3 (5)	−7.8 ± 0.8 (6)	−9.7 ± 0.5 (2)	−9.6 ± 0.3 (5)
NSP8–NSP13 binding mode II	−10.7 ± 1.9 (4)	43.4 ± 2.3 (6)	−8.8 ± 1.1 (5)	−9.2 ± 1.0 (4)	−9.4 ± 0.4 (4)
NSP8-N–NSP13	−11.5 ± 1.9 (3)	−61.1 ± 2.3 (1)	−11.4 ± 0.7 (1)	−9.6 ± 0.7 (3)	−10.4 ± 0.3 (1)
NSP8-N–NSP13 (7YWR)	−13.1 ± 1.9 (1)	−42.0 ± 2.3 (4)	−9.4 ± 0.9 (4)	−8.6 ± 1.2 (6)	−9.9 ± 0.3 (2)
NSP8-C–NSP13	−13.0 ± 1.9 (2)	−55.3 ± 2.3 (3)	−10.3 ± 1.0 (2)	−10.8 ± 1.5 (1)	−9.8 ± 0.4 (3)
NSP13–TBK1	−8.1 ± 1.9 (6)	−57.7 ± 2.3 (2)	−9.8 ± 0.9 (3)	−8.7 ± 0.6 (5)	−9.8 ± 0.3 (3)

Notably, the NSP8-N fragment binds NSP13 with higher affinity (𝛥*G* = −11.5 kcal/mol) than either RTC binding mode. This result supports the structural observation that NSP8-N engages a broader and more polar–electrostatic surface on NSP13, including residues involved in host factor recognition. The binding to the experimentally resolved NSP8-N (PDB ID 7YWR) demonstrated the best binding affinity in our model. In contrast, the C-terminal fragment of NSP8 shows one of the strongest predicted binding to NSP13 (𝛥*G* = −13.0 kcal/mol), reflecting its extensive hydrophobic and polar contact network; however, as shown by structural analysis, this interaction occurs at a surface distinct from the TBK1-binding interface and is therefore not expected to be competitive.

In comparison, the NSP13–TBK1 complex displays a significantly weaker binding free energy (𝛥*G* = −8.09 kcal/mol) than all NSP8-derived complexes. This difference translates into a markedly higher dissociation constant and lower affinity, indicating that viral NSP8-derived binders could potentially outcompete TBK1 for NSP13 binding when present as observed while taking into account the approximations made with the employed binding energy estimation method. *K*_d_ values are estimated under standard conditions and should be interpreted qualitatively.

We compared our results to 4 other external estimators—MM/PBSA, PRODIGY, PBEE, and PPR-Affinity (Table 2, columns 3 to 6). These calculations were based on all conformations of the respective complex in the largest cluster of the ensemble. Because the different predictors are based on different assumptions and scales, the safest interpretation is to compare ranks rather than absolute 𝛥*G_bind_* values (Table 3).

**Table 3.** Ranking of the evaluated protein–protein complexes

Complex	Ranks	Mean rank
NSP8-N–NSP13	3, 1, 1, 3, 1	1.8
NSP8-C–NSP13	2, 3, 2, 1, 3	2.2
NSP8-N–NSP13 (7YWR)	1, 4, 4, 6, 2	3.4
NSP13–TBK1	6, 2, 3, 5, 3	3.8
NSP8–NSP13 binding mode I	5, 5, 6, 2, 5	4.6
NSP8–NSP13 binding mode II	4, 6, 5, 4, 4	4.6

Using the ranks across all 5 methods, the most consistent signal is that NSP8-N–NSP13 and NSP8-C–NSP13 form the top tier. NSP8-N–NSP13 has the best mean rank, approximately 1.8, and is ranked first by MM/PBSA, PRODIGY, and PPB. NSP8-C–NSP13 follows closely, with a mean rank of approximately 2.2, and is ranked within the top 3 by every method. Thus, despite differences in predicted 𝛥*G_bind_* values, these 2 complexes appear to represent the most favorable binding arrangements among the investigated systems.

NSP13–TBK1 occupies an intermediate position: It is favored by MM/PBSA, PRODIGY, and PPB, but receives poor ranks from CNN and PBEE. The 2 full-length NSP8–NSP13 binding modes are generally less favorable, with binding mode II consistently ranked in the lower half by all methods and binding mode I supported mainly by PBEE but disfavored by the other predictors. Overall, the rank consensus supports the qualitative ordering NSP8-N–NSP13 ≈ NSP8-C–NSP13 > NSP8-N–NSP13 (7YWR)/NSP13–TBK1 > NSP8–NSP13 binding modes I and II, with the clearest conclusion being that the N- and C-terminal NSP8 fragments provide more favorable NSP13-binding solutions than the 2 full-length binding modes.

This conclusion should be framed as a consensus computational ranking, not as a definitive thermodynamic ordering. The spread among predictors is expected because protein–protein binding free-energy prediction remains challenging, with modern resources such as PPB-Affinity emphasizing that data availability and model generalization are still limiting factors for artificial intelligence-based affinity prediction. Therefore, the most defensible interpretation is to prioritize NSP8-N–NSP13 and NSP8-C–NSP13 for further structural or experimental validation.

These results establish a clear energetic hierarchy among the competing complexes. While full-length NSP8 interactions stabilize NSP13 within the RTC, the NSP8-N fragment exhibits sufficient affinity to displace TBK1 from NSP13, providing a quantitative thermodynamic basis for the experimentally observed restoration of IFN-β signaling upon NSP8-N coexpression.

### Experimental results

#### NSP13 and NSP8 modulate IFN-β production in response to cytosolic dsRNA

To induce IFN-I signaling, A549 cells were stimulated with poly(I:C), a synthetic analog of dsRNA that activates the cytosolic pattern-recognition receptors RIG-I and MDA5, thereby mimicking viral RNA recognition. Cells were transfected with poly(I:C) together with expression plasmids encoding viral proteins and/or an EV control. IFN-β transcription was quantified by RT-qPCR and normalized to the EV condition.

**Fig. 6.**
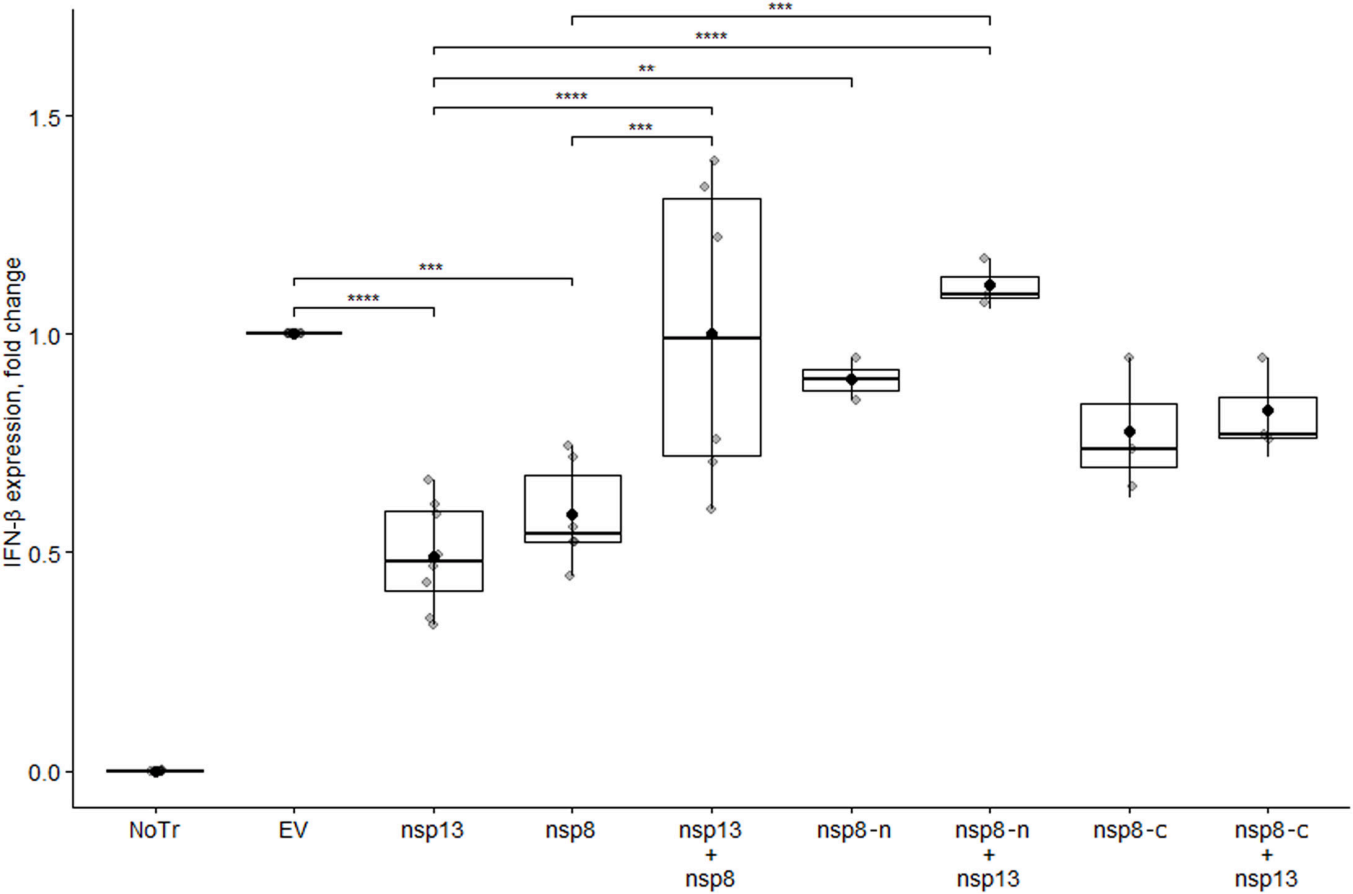
Influence of NSP13 and NSP8 on the IFN-β response to poly(I:C). A549 cells were stimulated with 2 μg/ml poly(I:C), except for the negative control group (NoTr). Cells were transfected with EV, plasmids expressing NSP13, NSP8, the N-terminal domain (NSP8-N), or the C-terminal domain (NSP8-C) of NSP8, as well as combinations of these constructs, with EV used to equalize total plasmid amounts. IFN-β mRNA levels were quantified by RT-qPCR and normalized to GAPDH. Data are presented as fold change relative to EV control and represent mean ± SD from 3 or more independent experiments. Statistical analysis was performed using one-way ANOVA with Tukey’s post hoc test. **P < 0.01; ***P < 0.001; ****P < 0.0001. Nonsignificant differences are not indicated.

Expression of either NSP13 or NSP8 alone resulted in a significant reduction of poly(I:C)-induced IFN-β expression compared to the EV control (Fig. 6), confirming that both viral proteins independently suppress IFN-I signaling. The calculated *P* values for all pairs of conditions are reported in Table S1. In contrast, coexpression of NSP8 and NSP13 led to a marked restoration of IFN-β expression, reaching levels comparable to those observed in EV-transfected cells (Fig. 6). This reversal of IFN-β inhibition suggests that the interaction between NSP8 and NSP13 counteracts their individual inhibitory effects on innate immune signaling.

Importantly, this functional outcome is consistent with our MD simulations, which indicate that NSP8 can bind NSP13 at defined interfaces with high affinity. In particular, the N-terminal region of NSP8 was predicted to occupy a surface of NSP13 that overlaps with the TBK1-binding interface, thereby potentially preventing NSP13 from engaging host signaling components required for interferon suppression.

#### The N-terminal domain of NSP8 prevents the inhibition of IFN-β caused by NSP13

Structural studies of the SARS-CoV-2 replication–transcription complex have shown that NSP13 interacts with NSP8 via the ZBD of NSP13 and the long N-terminal α-helix of NSP8. Guided by our simulation results, which predict a strong and competitive interaction between the N-terminal region of NSP8 and NSP13, we investigated whether this domain alone is sufficient to reverse NSP13-mediated interferon suppression.

To this end, the original NSP8 expression construct was divided into 2 separate plasmids encoding either the N-terminal domain (NSP8-N, residues 1 to 87) or the C-terminal domain (NSP8-C; residues 88 to 198). Coexpression of NSP13 with the NSP8-N resulted in a significant restoration of IFN-β expression compared to NSP13 alone (Fig. 6). In contrast, coexpression of NSP13 with the NSP8-C did not significantly alter the inhibitory effect of NSP13 on IFN-β production.

These results provide experimental support for the model derived from our MD simulations. Specifically, they indicate that the N-terminal domain of NSP8 is both necessary and sufficient to counteract NSP13-mediated inhibition of interferon signaling. We propose that binding of the NSP8-N α-helical region to NSP13 competitively displaces NSP13 from its cellular target TBK1, thereby relieving suppression of the IFN-β pathway.

## Discussion

A defining feature of many RNA viruses is their ability to suppress the host IFN-I response, thereby ensuring efficient replication and immune evasion. SARS-CoV-2 employs multiple NSPs to antagonize innate immunity, among which NSP13 plays a central role [15,52]. In addition to its essential helicase and RNA 5′-triphosphatase activities within the RTC, NSP13 suppresses IFN-β production through interaction with host signaling components such as TBK1 [15,16]. Because NSP13 is highly conserved and structurally central to viral replication [1,6,53], targeting its protein–protein interactions (PPIs) represents a mechanistically attractive antiviral strategy. However, direct enzymatic inhibition of NSP13 may be challenging due to its multifunctionality and structural integration within the RTC [10,54]. We therefore explored an alternative strategy: competitive modulation of NSP13 interactions using fragments derived from its natural viral partner, NSP8. This approach aims not to inhibit the catalytic core directly but to exploit structural hotspots required for NSP13 association with both viral and host factors.

Overall, the recent NSP13 literature strongly converges on the idea that SARS-CoV-2 helicase is a conserved, druggable antiviral target, but almost all studies approach it through small-molecule ligand discovery rather than protein–protein interaction disruption. The CACHE Challenge #2 [55] work benchmarked many computational and ML methods against the RNA-binding site of NSP13, asking teams to nominate drug-like ligands and then experimentally testing nearly 2,000 compounds, with a small but real fraction confirmed as binders; the FRASE-bot CACHE paper similarly targeted the RNA-binding groove and reported several micromolar binders after 2 prospective rounds, supporting the feasibility but also the difficulty of NSP13 hit finding. The FragmentScout/FragmentScout–LigandScout workflow mined crystallographic fragment data and identified 13 novel micromolar NSP13 inhibitors validated by antiviral cellular and ThermoFluor assays, while the ChemMedChem structure-based/docking/alchemical-simulation paper used a computational pipeline to nominate putative NSP13 inhibitors, with follow-up prioritization suggesting potent candidate ligands. Several 2022–2024 virtual-screening and repurposing studies reached similar conclusions from different chemical spaces: Conserved/druggable pockets have been found and micromolar inhibitors of NSP13 enzymatic activity have been suggested [56,57], although many of these remain computational predictions requiring stronger biochemical and antiviral validation [58,59]. Enzymatic NSP13 inhibition was found to translate into antiviral activity [60–63].

To the best of our knowledge, essentially all other teams are searching for small-molecule inhibitors, including drug-like ligands, fragments, repurposed drugs, and natural products. Here, we propose to use inhibitory proteins, peptides, or peptidomimetics, as therapeutic inhibitors. The guiding hypothesis of this work was that the N-terminal domain of NSP8 could bind NSP13 with sufficient affinity and spatial overlap to displace NSP13 from host immune regulators such as TBK1. By combining MD simulations, structural contact mapping, binding free energy prediction using a trained CNN, and functional interferon assays, we sought to test whether NSP8-derived fragments could act as competitive inhibitors of NSP13-mediated immune suppression.

Our structural analysis confirms that the NSP13–TBK1 interface is stabilized by a dense polar–electrostatic core involving residues such as Glu^244^, His^245^, Lys^394^, and Glu^353^ on NSP13 interacting with Arg^577^, Arg^576^, Tyr^580^, and Glu^575^ on TBK1. This interface is flanked by hydrophobic packing interactions, forming a stable regulatory complex consistent with previously reported suppression of IRF3 phosphorylation [15,16,64]. Comparative structural mapping revealed that the NSP8 N-terminal fragment occupies a partially overlapping surface on NSP13. Key TBK1-contact residues of NSP13, including Ser^350^ and Glu^353^, are also engaged by NSP8-N. The NSP8-N spans the ZBD-proximal corridor and extends toward the Ser^350^–Thr^351^ region, effectively covering a substantial portion of the TBK1-facing surface.

The observed dual binding profile (Fig. 1), anchoring within the ZBD and extending toward the central helicase domains, suggests that the NSP8-N peptide may act as a competitive binder capable of perturbing the structural configuration of NSP13. By occupying the ZBD and adjacent surfaces, it may hinder NSP13’s ability to integrate into the functional RTC complex or to interact with cellular proteins such as TBK1. This hypothesis is supported by the location of residues like Lys^345^ and Thr^351^, which lie within regions previously implicated in RTC interface formation and potential host interactions. This complex interaction map supports the hypothesis that the N-terminal segment of NSP8 forms a stable and functionally relevant interface with NSP13, providing a structural basis for competitive interference with alternative NSP13-binding partners such as NSP8 and TBK1.

Importantly, binding free energy estimation ranks the NSP13–TBK1 complex as energetically weaker than NSP8-N–NSP13 binding. This energetic hierarchy strongly supports the possibility of competitive displacement under coexpression conditions.

Functional assays in poly(I:C)-stimulated A549 cells demonstrate that both NSP13 and NSP8 individually suppress IFN-β production, consistent with previous reports that NSP13 inhibits TBK1 and IRF3 phosphorylation [16] and that NSP8 interferes with MDA5 activation [65]. Notably, coexpression of NSP13 with full-length NSP8 restored IFN-β levels to those of control cells, indicating attenuation of NSP13-mediated inhibition.

This restorative effect was replicated by coexpression of NSP13 with the N-terminal domain (NSP8-N) of NSP8, but not with its C-terminal domain. The C-terminal domain itself was previously found to suppress IFN-I induction upstream at the level of MDA5 (44), but the interaction with the MDA5 CARD domain was mediated by N2 (E32-D101), which is cleaved in our experiment. This could explain the suppression of IFN-I induction by nsp8 alone, but not by any of the fragments. Therefore, the nsp8 fragments no longer exert suppressive effects in our experiment, whereas the NSP8-N still blocks the suppression by nsp13. This is fully consistent with our structural and energetic data, which demonstrate that the N-terminal α-helical region forms the primary high-affinity interface with NSP13 and overlaps with the TBK1-binding surface. Together, the structural, energetic, and functional data converge on a model in which NSP8-N competes with TBK1 for binding to NSP13, thereby alleviating suppression of IFN-β signaling.

Beyond immune restoration, our data suggest a broader competitive mechanism affecting viral replication. Structural analysis revealed that NSP8-N occupies residues on NSP13 that are required for both canonical NSP8–NSP13 binding modes within the RTC. In binding mode I, NSP8-N engages Tyr^93^, Met^68^, and Phe^90^, residues essential for ZBD-mediated recruitment of NSP13. In binding mode II, it additionally interacts with Gly^67^ and Met^68^, which contribute to hydrophobic stabilization of the second NSP8–NSP13 pair. The shared involvement of Met^68^ in both RTC configurations is particularly notable.

Because NSP8-N exhibits a higher predicted binding affinity than either native RTC binding mode, the thermodynamic hierarchy favors NSP8-N engagement. These observations suggest that NSP8-N may sequester NSP13 in a nonproductive complex, thereby interfering not only with TBK1 binding but also with proper integration of NSP13 into the RTC.

The dual competitive capacity of NSP8-N, interfering with both immune evasion and RTC assembly, positions the N-terminal α-helical motif of NSP8 as a structurally informed template for inhibitor design. Stabilized α-helical peptides, peptidomimetics, or small molecules targeting this overlapping hotspot could exploit the conserved structural features of NSP13 [1,6], potentially maintaining efficacy across emerging variants.

## Conclusion

In this study, we combined structural modeling, MD simulations, neural network-based binding free energy estimation, and functional interferon assays to elucidate the competitive interaction landscape of SARS-CoV-2 NSP13. Our results provide a coherent framework linking structural interference at the molecular level with restoration of innate immune signaling at the cellular level.

We demonstrate that the N-terminal fragment of NSP8 (NSP8-N, residues 1 to 87) forms a stable and high-affinity complex with NSP13. Structural analyses reveal that NSP8-N occupies residues on NSP13 that are critically involved in 2 distinct functional contexts: (a) binding to the host kinase TBK1, which mediates suppression of IFN-I signaling, and (b) canonical recruitment of NSP13 into the RTC via dual NSP8-binding modes. In particular, residues such as Met^68^, Tyr^93^, Phe^90^, Gly^67^, Ser^350^, and Thr^351^ of NSP13, key contributors to both RTC anchoring and host interaction, are directly engaged by NSP8-N.

Free energy predictions further support a competitive hierarchy, showing that NSP8-N binds NSP13 with higher affinity than either of the 2 native NSP8–NSP13 RTC configurations and more strongly than the NSP13–TBK1 complex. This thermodynamic preference is consistent with the observed displacement effect in our cellular model, where coexpression of NSP13 with full-length NSP8 or specifically with its N-terminal domain restores IFN-β expression to near-control levels. These findings strongly suggest that NSP8-N sequesters NSP13 into a nonproductive complex, thereby limiting its availability for TBK1 binding and alleviating NSP13-mediated inhibition of innate immune signaling.

Beyond immune restoration, our structural comparison indicates that NSP8-N also occludes residues required for both canonical NSP8–NSP13 interaction modes within the RTC. This raises the possibility that high-affinity NSP8-derived fragments could interfere with proper incorporation of NSP13 into the viral replication machinery. Although further experimental validation is required to confirm direct effects on RTC assembly, our data support a dual-inhibition model targeting both immune evasion and viral replication.

Collectively, our integrative approach identifies the N-terminal α-helical segment of NSP8 as a structurally and functionally privileged motif capable of competitively modulating NSP13 interactions. These findings provide a rational basis for the development of NSP8-derived peptidomimetics or small molecules designed to selectively disrupt NSP13-mediated immune suppression while potentially impairing replication complex stability.

## Data Availability

All data supporting the findings of this study are included in this article and its Supplementary Materials. Any further requests for data should be directed to the corresponding authors.
