# Supplementary material for: SARS-CoV-2 NSP8-Derived Peptide Effectively Suppresses the Activity of Helicase NSP13
Source: Comput Struct Biotechnol J. 2026 Aug 3;35(1):0174. doi: 10.34133/csbj.0174 (PMC13429913; doi:10.34133/csbj.0174)
Supplement: Supplementary 1 — Figs. S1 to S10 Table S1 [file csbj.0174.f1.pdf]

Supplementary Material for
SARS-CoV-2 NSP8 derived peptide effectively suppresses the activity of helicase NSP13

Shina Pashova[a], Peicho Petkov[b,*], Rositsa Hristova[c], Elena Krachmarova[c], Genoveva Nacheva[c], Anastas Gospodinov[c], Elena Lilkova[d,e], Nevena Ilieva[d,e], Miroslav Rangelov[f], Nadezhda Todorova[g], Anastas Pashov[h,**] and Leandar Litov[b]

[a] *Institute of Biology and Immunology of Reproduction "Acad. K. Bratanov", Bulgarian Academy of Sciences, 73 Tsarigradsko shose Blvd., Sofia, 1113, Bulgaria*

[b] *Sofia University "St. Kl. Ohridsky", Physics Faculty, 5, James Bourchier Blvd, Sofia, 1164, Bulgaria*

[c] *Institute of Molecular Biology "Acad. Roumen Tsanev", Bulgarian Academy of Sciences, Acad. G. Bonchev Str., Block 21, Sofia, 1113, Bulgaria*

[d] *Institute of Information and Communication Technologies, Bulgarian Academy of Sciences, Acad. G. Bonchev Str., Block 25A, Sofia, 1113, Bulgaria*

[e] *Centre of Excellence in Informatics and Information and Communication Technologies, Acad. G. Bonchev Str., Block 25A, Sofia, 1113, Bulgaria*

[f] *Institute of Organic Chemistry with Centre of Phytochemistry, Bulgarian Academy of Sciences, Acad. G. Bonchev Str., Block 9, Sofia, 1113, Bulgaria*

[g] *Institute of Biodiversity and Ecosystem Research, Bulgarian Academy of Sciences, 2 Gagarin Str., Sofia, 1113, Bulgaria*

[h] *"Stephan Angeloff" Institute of Microbiology, Bulgarian Academy of Sciences, Acad. G. Bonchev Str., Block 26, Sofia, 1113, Bulgaria*

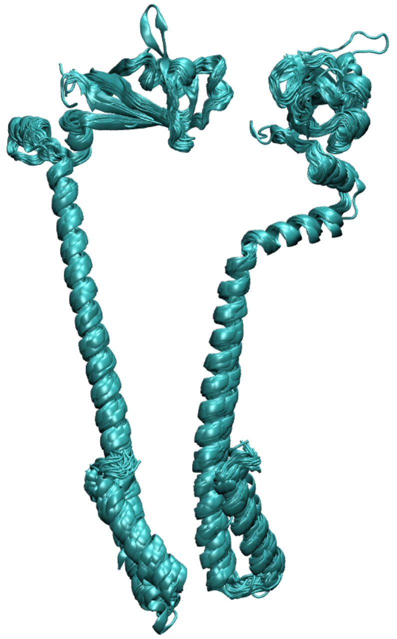

Fig. S1: Experimentally resolved NSP8 stuctures in the RTC. PDB IDs used: 6XEZ [1], 7C2K [8], 7CXM, 7CXN [15], 7DTE [9], 7ED5 [6], 7EGQ [14], 7KRN, 7KRO, 7KRP [4], 7RDX, 7RDY, 7RDZ, 7RE0, 7RE1, 7RE2, 7RE3, 7RE3, 7THM, 7UO4, 7UO7, 7UO9, 7UOB, 7UOE [5], 8GW1, 8GWB, 8GWE, 8GWF, 8GWG, 8GWI, 8GWK, 8GWM, 8GWN, 8GWO [12], 8SQ9, 8SQJ, 8SQK [7], 8XCH, 9IMK, 9IMM [13] , 9BLF [11], 9CPO [2], 9IKZ [3], 9PYW, 9PYZ, 9PZ0 [10], 9UHT

*Corresponding author
**Corresponding author
Email addresses: peicho@phys.uni-sofia.bg (P. Petkov); a_pashov@microbio.bas.bg (A. Pashov)
ORCID(s): 0000-0001-9857-2872 (S. Pashova); 0000-0002-0420-9480 (P. Petkov); 0000-0001-8362-5396 (R. Hristova); 0000-0002-9501-0724 (E. Krachmarova); 0000-0001-5663-8589 (G. Nacheva); 0000-0001-6216-9563 (A. Gospodinov); 0000-0003-2008-710X (E. Lilkova); 0000-0002-2372-2319 (N. Ilieva); 0000-0002-7953-7593 (M. Rangelov); 0000-0002-7195-195X (N. Todorova); 0000-0002-6033-3566 (A. Pashov); 0000-0002-8511-6883 (L. Litov)

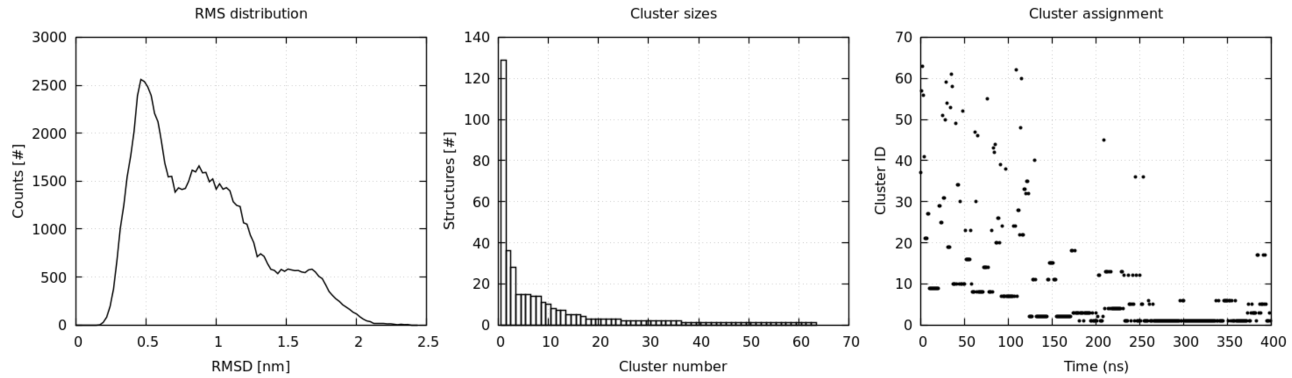

(a) NSP13–NSP8-N, cutoff = 0.4 nm

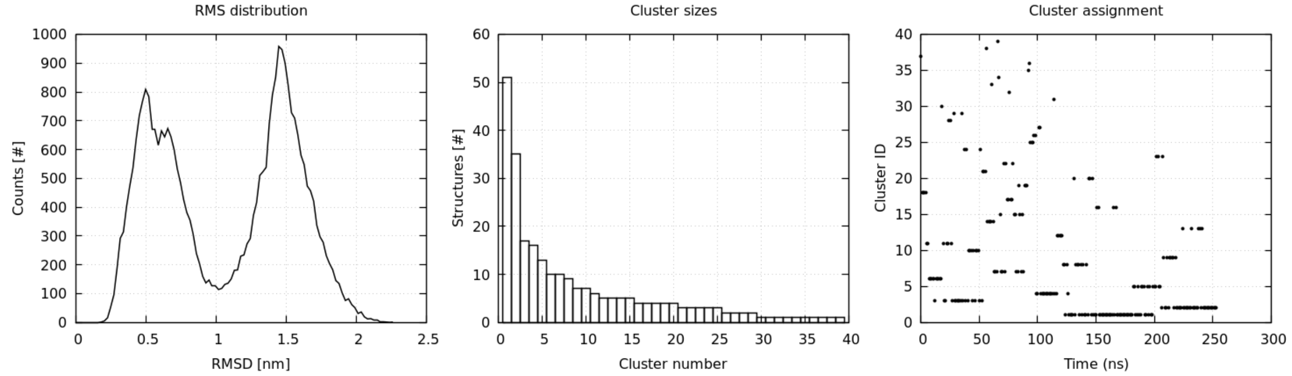

(b) NSP13–NSP8-N (7YWR), cutoff = 0.4 nm

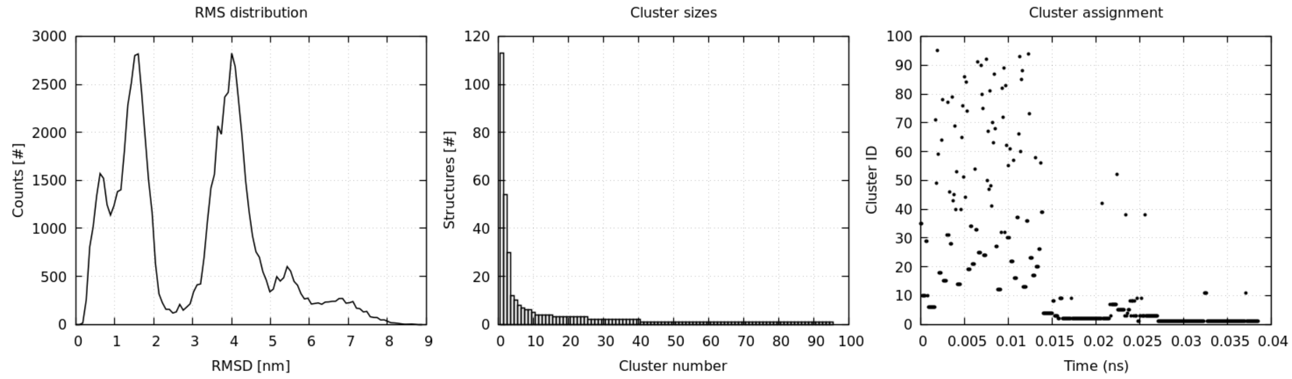

(c) NSP13–NSP8-C), cutoff = 0.8 nm

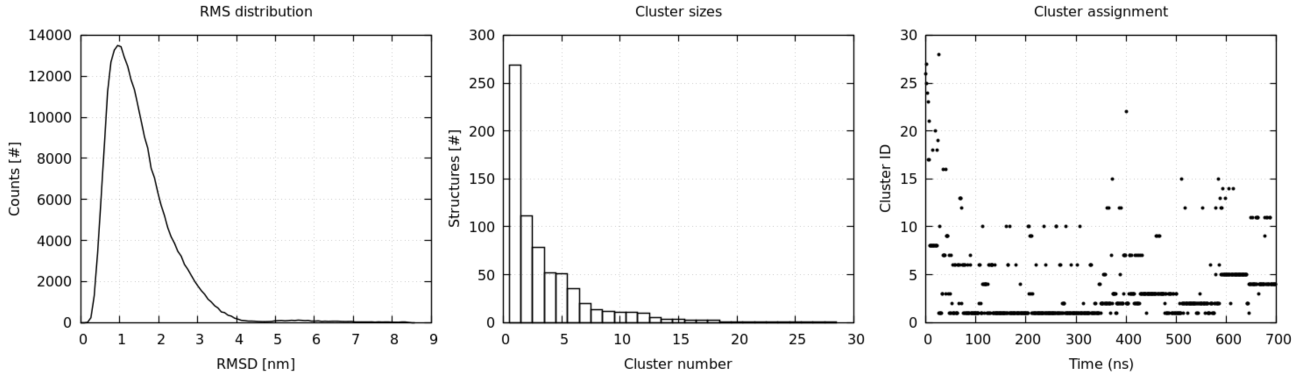

(d) NSP13–TBK1), cutoff = 0.8 nm

Fig. S2: Clustering data for NSP13 complexes.

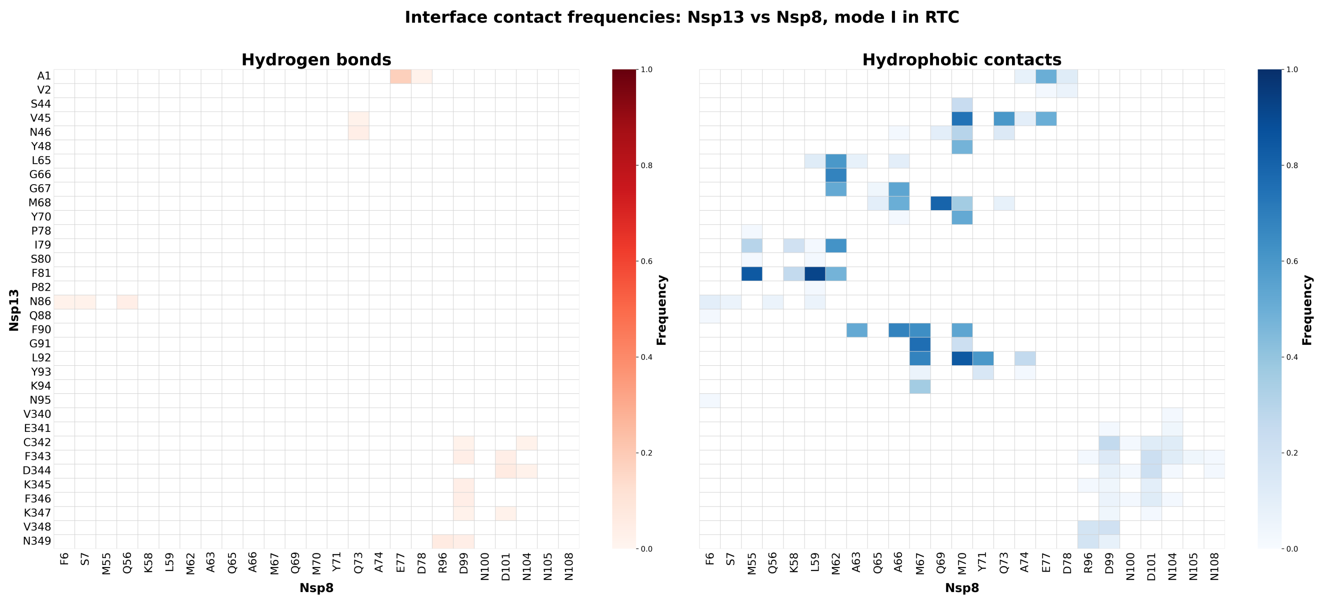

Fig. S3: Contact maps of the NSP13–NSP8 complex, binding mode I in the RTC.

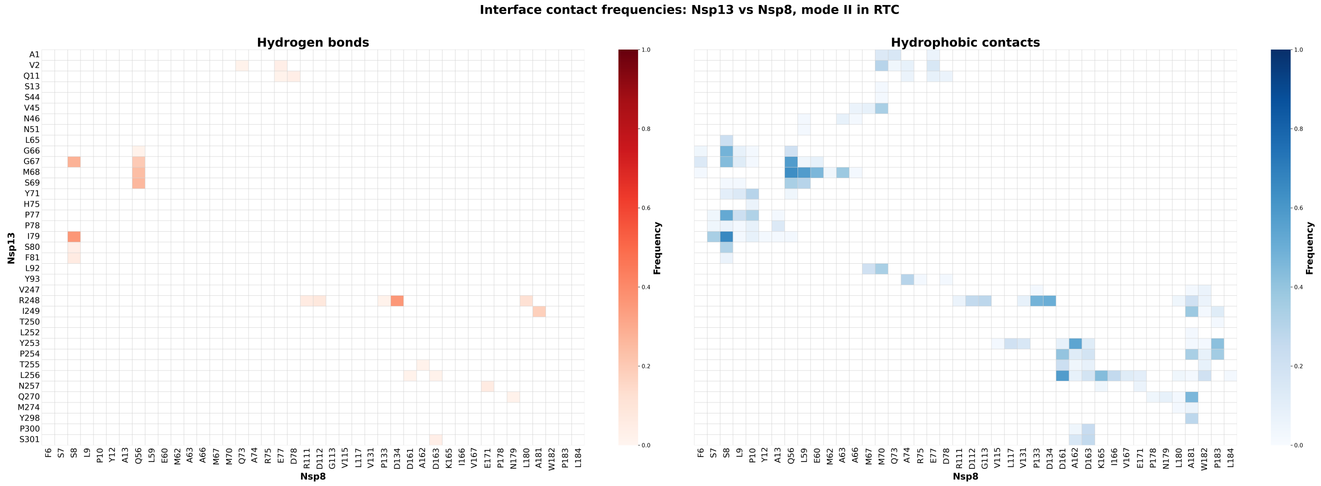

Fig. S4: Contact maps of the NSP13–NSP8 complex, binding mode II in the RTC.

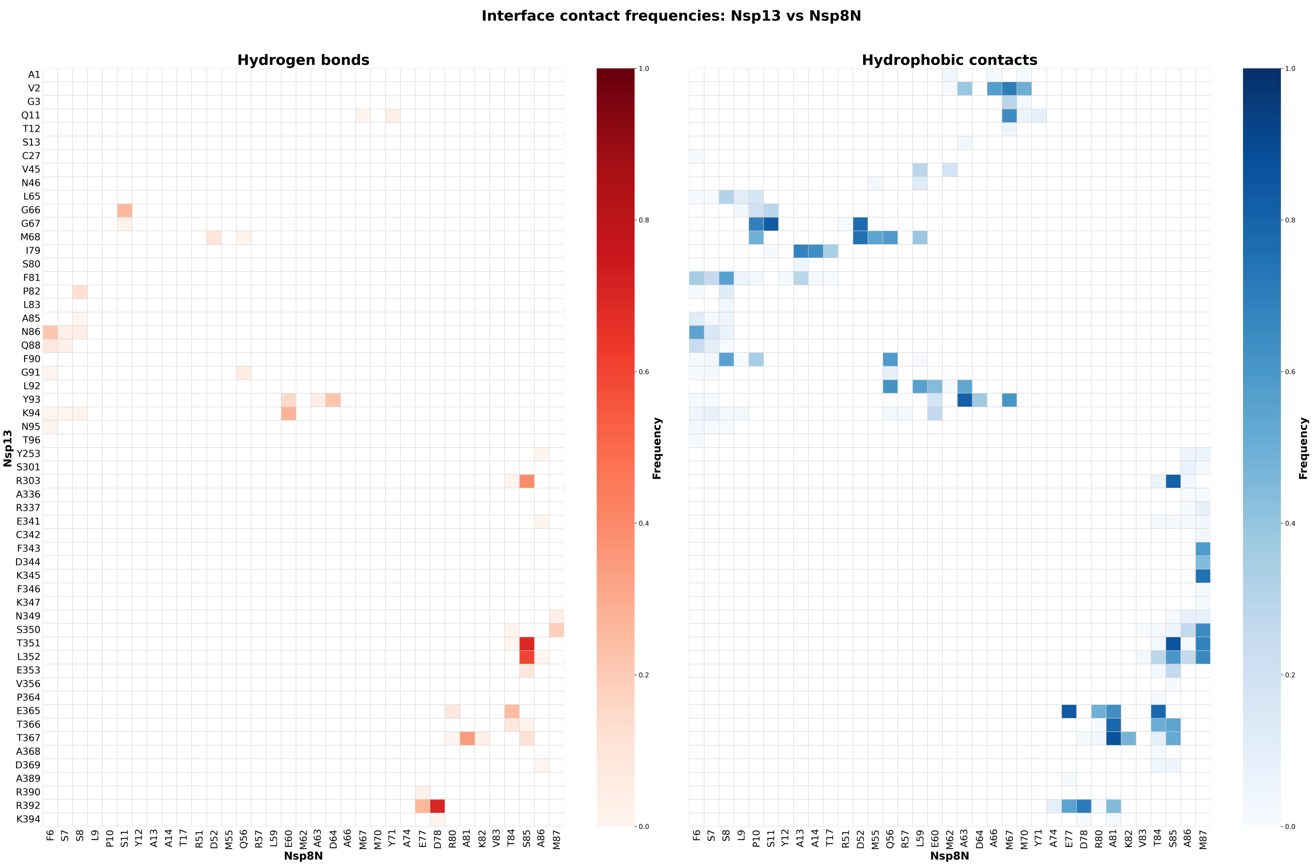

Fig. S5: Contact maps of the NSP13–NSP8-N complex.

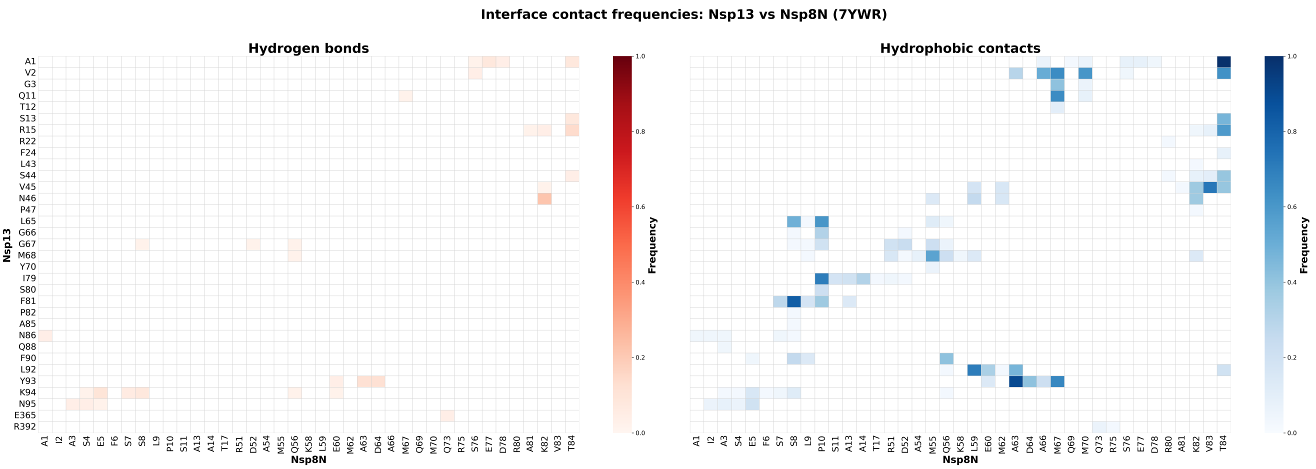

Fig. S6: Contact maps of the NSP13–NSP8-N (7YWR) complex.

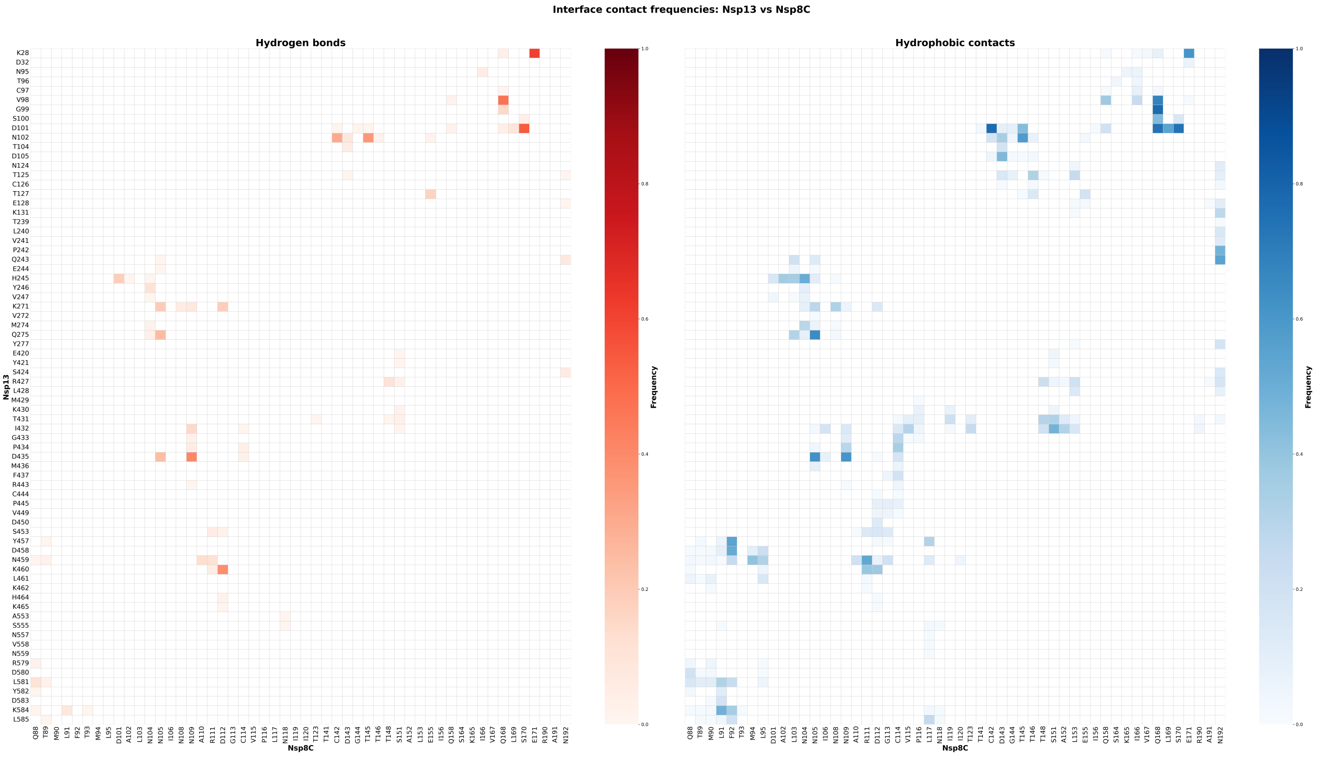

Fig. S7: Contact maps of the NSP13–NSP8-C complex.

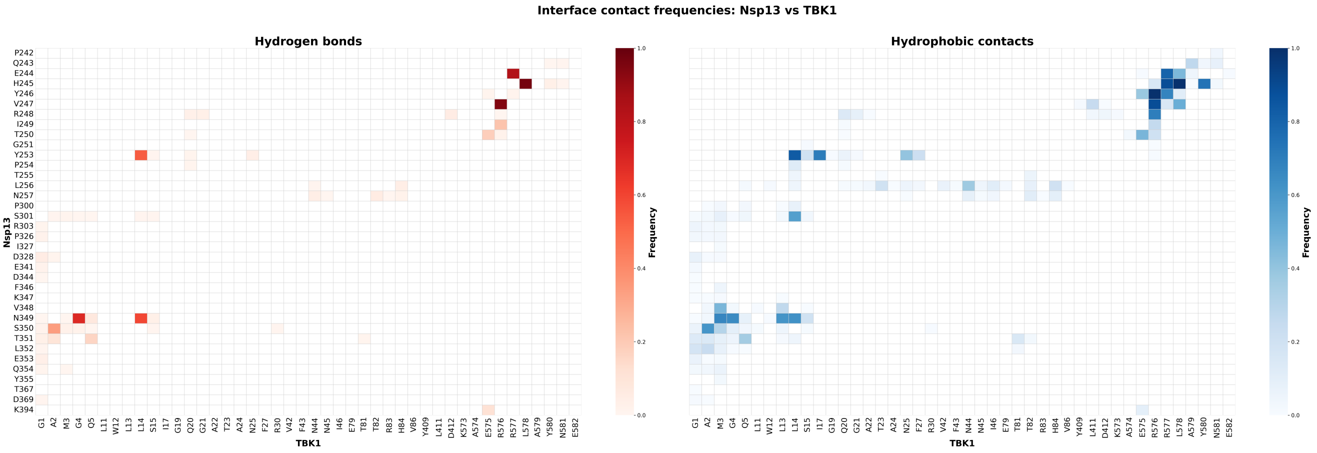

Fig. S8: Contact maps of the NSP13–TBK1 complex.

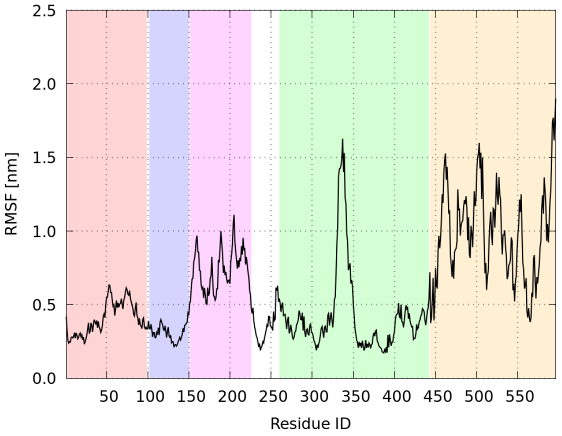

(a) Mode I in the RTC

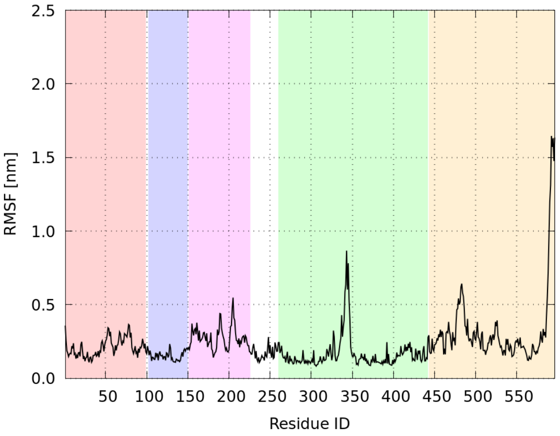

(b) Mode II in the RTC

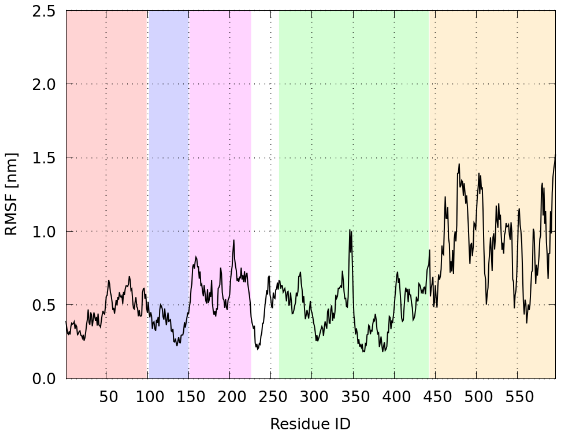

(c) NSP13–NSP8-N

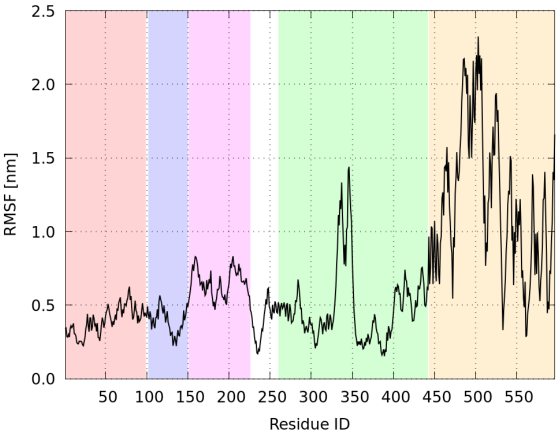

(d) NSP13–NSP8-N (7YWR)

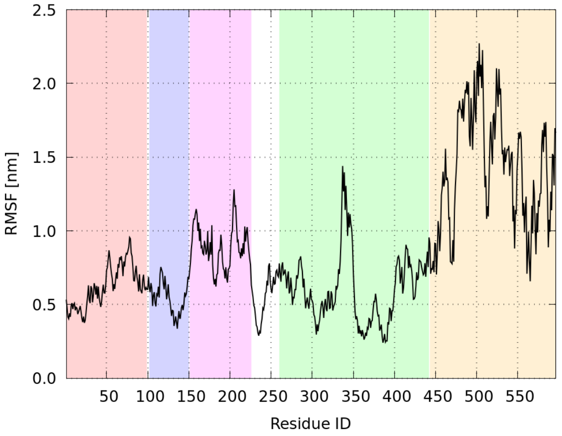

(e) NSP13–NSP8-C

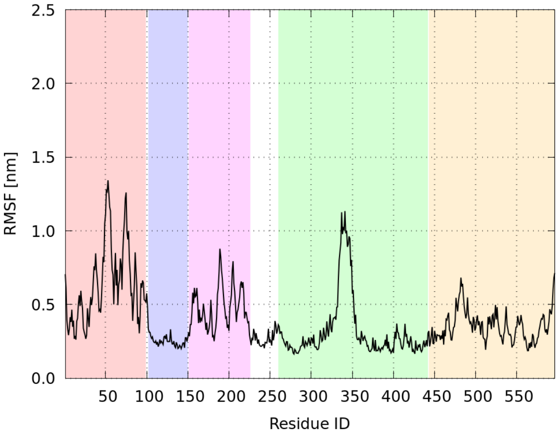

(f) NSP13–TBK1

Fig. S9: RMSF of NSP13. The NSP13 protein domains are colored as follows: ZBD (aa 1 – 99) in red; Stalk (aa 102 – 150) in blue; 1B (aa 151–229) in purple; Rec1A (aa 260 – 442) in green; Rec2A (aa 443 – 596) in orange; and loops in white.

Table S1
Pairwise p-values using one-way ANOVA with Tukey's post hoc test.

	NoTr	EV	NSP13	NSP8	NSP13 +NSP8	NSP8-N NSP8-N	NSP13+ NSP8-N	NSP8-C NSP8-C	NSP13+ NSP8-C
NoTr									
EV			0.000001	0.000275	1	0.981894	0.970117	0.431609	0.72205
NSP13		0.000001		0.959913	0.000006	0.007767	0.000012	0.143248	0.051266
NSP8		0.000275	0.959913		0.000691	0.111836	0.000438	0.675974	0.39849
NSP13+NSP8		1	0.000006	0.000691		0.983882	0.980058	0.474382	0.751336
NSP8-N		0.981894	0.007767	0.111836	0.983882		0.707541	0.986266	0.999537
NSP13+NSP8-N		0.970117	0.000012	0.000438	0.980058	0.707541		0.170161	0.336784
NSP8-C		0.431609	0.143248	0.675974	0.474382	0.986266	0.170161		0.999985
NSP13+NSP8-C		0.722050	0.051266	0.398490	0.751336	0.999537	0.336784	0.999985	

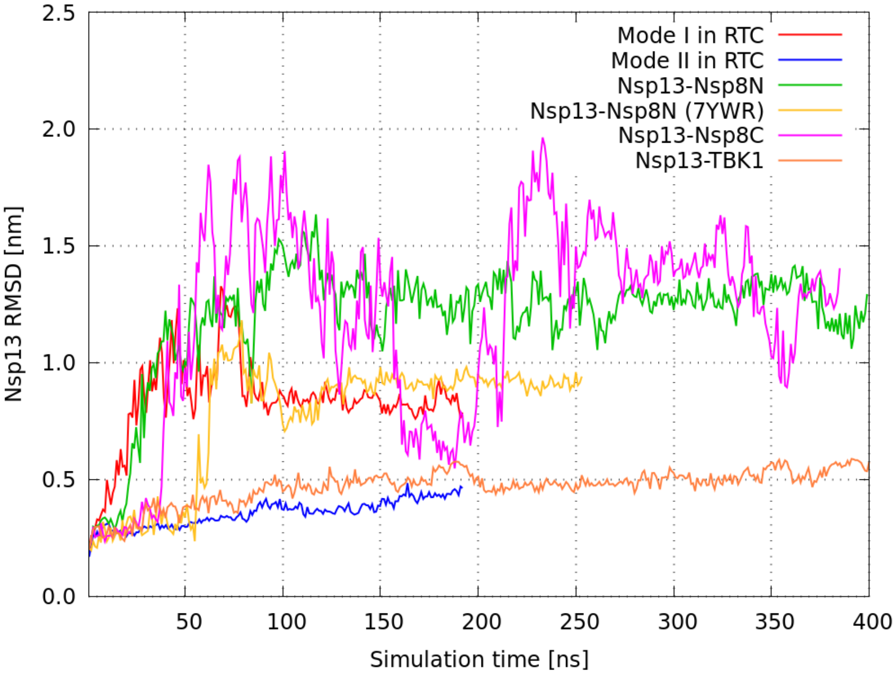

Fig. S10: RMSD of NSP13.

References

[1] Chen, J., Malone, B., Llewellyn, E., Grasso, M., Shelton, P.M.M., Olinares, P.D.B., Maruthi, K., Eng, E.T., Vatandaslar, H., Chait, B.T., Kapoor, T.M., Darst, S.A., Campbell, E.A., 2020. Structural basis for helicase-polymerase coupling in the sars-cov-2 replication-transcription complex. Cell 182, 1560–1573.e13. doi:10.1016/j.cell.2020.07.033.

[2] Hoferle, P.J., Anderson, T.K., Kirchdoerfer, R.N., 2024. A genus-specific nsp12 region impacts polymerase assembly in alphacoronavirus and gammacoronavirus. Journal of Biological Chemistry 300, 107802. doi:10.1016/j.jbc.2024.107802.

[3] Huang, Y., Tan, L., Liu, Y., Zhao, H., Wang, J., Ge, J., Ye, S., Liu, Z., Lan, W., Huang, B., Zhang, H., Gao, Y., Yan, L., Rao, Z., Lou, Z., 2025. Response to: The mechanism for gtp-mediated rna capping by the SARS-CoV-2 NiRAN domain remains unresolved. Cell 188, 4462–4469.e9. doi:10.1016/j.cell.2025.05.045.

[4] Malone, B., Chen, J., Wang, Q., Llewellyn, E., Choi, Y.J., Olinares, P.D.B., Cao, X., Hernandez, C., Eng, E.T., Chait, B.T., Shaw, D.E., Landick, R., Darst, S.A., Campbell, E.A., 2021. Structural basis for backtracking by the sars-cov-2 replication–transcription complex. Proceedings of the National Academy of Sciences 118, e2102516118. doi:10.1073/pnas.2102516118.

[5] Malone, B.F., Perry, J.K., Olinares, P.D.B., Lee, H.W., Chen, J., Appleby, T.C., Feng, J.Y., Bilello, J.P., Ng, H., Sotiris, J., Ebrahim, M., Chua, E.Y.D., Mendez, J.H., Eng, E.T., Landick, R., Götte, M., Chait, B.T., Campbell, E.A., Darst, S.A., 2023. Structural basis for substrate selection by the sars-cov-2 replicase. Nature 614, 781–787. doi:10.1038/s41586-022-05664-3.

[6] Shannon, A., Fattorini, V., Sama, B., Selisko, B., Feracci, M., Falcou, C., Gauffre, P., El Kazzi, P., Delpal, A., Decroly, E., Alvarez, K., Eydoux, C., Guillemot, J.C., Moussa, A., Good, S.S., La Colla, P., Lin, K., Sommadossi, J.P., Zhu, Y., Yan, X., Shi, H., Ferron, F., Canard, B., 2022. A dual mechanism of action of at-527 against sars-cov-2 polymerase. Nature Communications 13, 621. doi:10.1038/s41467-022-28113-1.

[7] Small, G.I., Fedorova, O., Olinares, P.D.B., Chandanani, J., Banerjee, A., Choi, Y.J., Molina, H., Chait, B.T., Darst, S.A., Campbell, E.A., 2023. Structural and functional insights into the enzymatic plasticity of the SARS-CoV-2 NiRAN domain. Molecular Cell 83, 3921–3930.e7. doi:10.1016/j.molcel.2023.10.001.

[8] Wang, Q., Wu, J., Wang, H., Gao, Y., Liu, Q., Mu, A., Ji, W., Yan, L., Zhu, Y., Zhu, C., Fang, X., Yang, X., Huang, Y., Gao, H., Liu, F., Ge, J., Sun, Q., Yang, X., Xu, W., Liu, Z., Yang, H., Lou, Z., Jiang, B., Guddat, L.W., Gong, P., Rao, Z., 2020. Structural basis for rna replication by the sars-cov-2 polymerase. Cell 182, 417–428.e13. doi:10.1016/j.cell.2020.05.034.

[9] Wu, J., Wang, H., Liu, Q., Li, R., Gao, Y., Fang, X., Zhong, Y., Wang, M., Wang, Q., Rao, Z., Gong, P., 2021. Remdesivir overcomes the s861 roadblock in sars-cov-2 polymerase elongation complex. Cell Reports 37, 109882. doi:10.1016/j.celrep.2021.109882.

[10] Xiao, Z., Das, A., Jain, A., Anderson, T.K., Cameron, C.E., Arnold, J.J., Dulin, D., Kirchdoerfer, R.N., 2025. The 2'-endo conformation of arabinose-ctp and arabinose-utp inhibit viral polymerases by inducing long pauses. bioRxiv doi:10.1101/2025.08.26.672356.

[11] Xiao, Z., Das, A., Jain, A., Anderson, T.K., Cameron, C.E., Arnold, J.J., Dulin, D., Kirchdoerfer, R.N., 2026. Incorporation of arabinose-ctp and arabinose-utp inhibits viral polymerases by inducing long pauses. Journal of Biological Chemistry 302, 111027. doi:10.1016/j.jbc.2025.111027.

[12] Yan, L., Huang, Y., Ge, J., Liu, Z., Lu, P., Huang, B., Gao, S., Wang, J., Tan, L., Ye, S., Yu, F., Lan, W., Xu, S., Zhou, F., Shi, L., Guddat, L.W., Gao, Y., Rao, Z., Lou, Z., 2022. A mechanism for sars-cov-2 rna capping and its inhibition by nucleotide analog inhibitors. Cell 185, 4347–4360.e17. doi:10.1016/j.cell.2022.09.037.

[13] Yan, L., Huang, Y., Liu, Y., Ge, J., Gao, S., Tan, L., Liu, L., Liu, Z., Ye, S., Wang, J., Xiong, J., Zhou, Y., Zhao, H., Zhao, X., Guddat, L.W., Gao, Y., Zhu, L., Rao, Z., Lou, Z., 2025. Structural basis for the concurrence of template recycling and rna capping in SARS-CoV-2. Cell 188, 7194–7205.e10. doi:10.1016/j.cell.2025.09.022.

[14] Yan, L., Yang, Y., Li, M., Zhang, Y., Zheng, L., Ge, J., Huang, Y.C., Liu, Z., Wang, T., Gao, S., Zhang, R., Huang, Y.Y., Guddat, L.W., Gao, Y., Rao, Z., Lou, Z., 2021. Coupling of n7-methyltransferase and 3′–5′ exoribonuclease with sars-cov-2 polymerase reveals mechanisms for capping and proofreading. Cell 184, 3474–3485.e11. doi:10.1016/j.cell.2021.05.033.

[15] Yan, L., Zhang, Y., Ge, J., Zheng, L., Gao, Y., Wang, T., Jia, Z., Wang, H., Huang, Y., Li, M., Wang, Q., Rao, Z., Lou, Z., 2020. Architecture of a sars-cov-2 mini replication and transcription complex. Nature Communications 11, 5874. doi:10.1038/s41467-020-19770-1.
